# Glutamine-Fructose-6-Phosphate Transaminase 2 (GFPT2) Is Upregulated in Breast Epithelial–Mesenchymal Transition and Responds to Oxidative Stress

**DOI:** 10.1016/j.mcpro.2021.100185

**Published:** 2021-12-17

**Authors:** Qiong Wang, Sigurdur Trausti Karvelsson, Aristotelis Kotronoulas, Thorarinn Gudjonsson, Skarphedinn Halldorsson, Ottar Rolfsson

**Affiliations:** 1 Center for Systems Biology, Biomedical Center, Faculty of Medicine, School of Health Sciences, University of Iceland, Reykjavik, Iceland; 2 Stem Cell Research Unit, Biomedical Center, Department of Anatomy, Faculty of Medicine, School of Health Sciences, University of Iceland, Reykjavík, Iceland

**Keywords:** proteomics, EMT, GFPT2, oxidative stress, claudin-low breast cancer, AKAP12, A-kinase anchor protein 12, AKR1B1, Aldo-keto reductase family 1, member B1 (aldose reductase), isoform CRA_a, AKR1C1, Aldo-keto reductase family 1, member C1, ALDH1A3, Aldehyde dehydrogenase 1 family, member A3, isoform CRA_a, ANXA3, Annexin A3, BP, Biological process, BRENCs, Breast endothelial cells, CADM3, Cell adhesion molecule 3, CCLE, Cancer Cell Line Encyclopedia, CDH1, Cadherin-1, CDH2, Cadherin-2, CID, Collision-induced dissociation, CTGF, Connective tissue growth factor, DCD, Dermicidin, DDA, Data-dependent acquisition, DSP, Desmoplakin, DTT, Dithiothreitol, EDTA, Ethylenediaminetetraacetic acid, EGF, Epidermal growth factor, EMT, Epithelial-mesenchymal transition, EPCAM, Epithelial cell adhesion molecule, ERBB2 (HER2), Erb-B2 receptor tyrosine kinase 2, ERK/MAPK, Mitogen-activated protein kinase, FASP, Filter-aided sample preparation, FBS, Fetal bovine serum, FDR, False discovery rate, FGFBP1, Fibroblast growth factor-binding protein 1, FLNC, Filamin-C, GALE, UDP-glucose 4-epimerase, GALNT7, N-acetylgalactosaminyltransferase 7, GES, Gene expression studies, GFPT1, Glutamine-fructose-6-phosphate aminotransferase 1, GFPT2, Glutamine-fructose-6-phosphate transaminase 2, GlcNAc-P, N-acetylglucosamine phosphate, GLUT1, Glucose transporter 1, GLUT4, Glucose transporter type 4, GO, Gene ontology, GSH, Reduced glutathione, GSK3-β, Glycogen synthase kinase 3 beta, GSSG, Oxidized glutathione, H_2_O_2_, Hydrogen peroxide, H_2_S, Hydrogen sulfide, HBP, Hexosamine biosynthesis pathway, HER2 (ERBB2), Human epidermal growth factor receptor 2, HMS LINCS, Harvard Medical School Library of Integrated Network-based Cellular Signatures, HPDL, 4-hydroxyphenylpyruvate dioxygenase-like protein, HUVECs, Human umbilical vein endothelial cells, IAA, Iodoacetamide, iBAQ, Intensity-based absolute quantification, IGF, Insulin like growth factor, IGF1R, Insulin like growth factor 1 receptor, IL18, Interleukin-18, IPA, Ingenuity Pathway Analysis, ITGA6, Integrin subunit alpha 6, ITGB4, Integrin subunit alpha 4, K5/6/8/14/19, Keratin 5/6/8/14/19, KEGG, Kyoto Encyclopedia of Genes and Genomes, KRAS, KRAS proto-oncogene, GTPase, KRT1, Keratin 1, LAD1, Ladinin-1, LAMA3, Laminin subunit alpha 3, LAMB3, Laminin subunit beta 3, LFQ, Label-free quantification, LKB1, Serine/threonine kinase 11 (STK11), MGST1, Microsomal glutathione S-transferase 1, MMS, Mesenchymal metabolic signature, MYL9, Myosin light chain 9, NDRG1, N-Myc downstream regulated 1, NF-κB, Nuclear factor kappa B, NSCLC, Non-small-cell lung cancer, NT5E, 5′-Nucleotidase Ecto, ODC, Ornithine decarboxylase, OGT, O-Linked N-Acetylglucosamine (GlcNAc) Transferase, PBS, Phosphate-buffered saline, PCSK1N, Proprotein convertase subtilisin/kexin type 1 Inhibitor, PFA, Paraformaldehyde, PGM2L1, Glucose 1,6-bisphosphate synthase, PGM3, Phosphoacetylglucosamine mutase, PKCα, Protein kinase C alpha, PKP2, Plakophilin-2, POMC, Pro-opiomelanocortin, PPP, Pentose phosphate pathway, PRSS23, Serine protease 23, PVDF, Polyvinylidene fluoride, PYGB, Glycogen phosphorylase, brain form, RCN3, Reticulocalbin 3, RELA, RELA Proto-Oncogene, NF-κB subunit, transcription factor p65, RT, Room temperature, RTK, Receptor tyrosine kinase, RT-qPCR, Quantitative reverse transcription PCR, S100A14, S100 calcium binding protein A14, S100A2, S100 calcium binding protein A2, SD, Standard deviation, SDS, Sodium dodecyl sulfate, SERPINB5, Serpin family B member 5, SERPINE1, Serpin family E member 1, SILAC, Stable isotope labeling by amino acids in cell culture, SIRT6, Sirtuin 6, SLC2A4, Solute carrier family 2 member 4, SLP-2, Stomatin-like protein 2, SQOR, Sulfide:quinone oxidoreductase, STRING, Search Tool for the Retrieval of Interacting Genes/Proteins, sXBP1, Spliced X-box binding protein 1, TAGLN, Transgelin, TCA, Tricarboxylic acid cycle, TCGA, The Cancer Genome Atlas, TFA, Trifluoroacetic acid, TGF-β, Transforming growth factor beta, TNFα, Tumor necrosis factor alpha, TWIST, Twist family BHLH transcription factor 1, UAP1, UDP-N-acetylhexosamine pyrophosphorylase, UDP, Uridine diphosphate, UDP-Glc, UDP-glucose, UDP-GlcA, UDP-glucuronate, UDP-GlcNAc, UDP-N-acetylglucosamine, UGDH, UDP-glucose 6-dehydrogenase, UTP, Uridine-5′-triphosphate, VIM, Vimentin

## Abstract

Breast cancer cells that have undergone partial epithelial–mesenchymal transition (EMT) are believed to be more invasive than cells that have completed EMT. To study metabolic reprogramming in different mesenchymal states, we analyzed protein expression following EMT in the breast epithelial cell model D492 with single-shot LFQ supported by a SILAC proteomics approach. The D492 EMT cell model contains three cell lines: the epithelial D492 cells, the mesenchymal D492M cells, and a partial mesenchymal, tumorigenic variant of D492 that overexpresses the oncogene HER2. The analysis classified the D492 and D492M cells as basal-like and D492HER2 as claudin-low. Comparative analysis of D492 and D492M to tumorigenic D492HER2 differentiated metabolic markers of migration from those of invasion. Glutamine-fructose-6-phosphate transaminase 2 (GFPT2) was one of the top dysregulated enzymes in D492HER2. Gene expression analysis of the cancer genome atlas showed that GFPT2 expression was a characteristic of claudin-low breast cancer. siRNA-mediated knockdown of *GFPT2* influenced the EMT marker vimentin and both cell growth and invasion *in vitro* and was accompanied by lowered metabolic flux through the hexosamine biosynthesis pathway (HBP). Knockdown of *GFPT2* decreased cystathionine and sulfide:quinone oxidoreductase (SQOR) in the transsulfuration pathway that regulates H_2_S production and mitochondrial homeostasis. Moreover, GFPT2 was within the regulation network of insulin and EGF, and its expression was regulated by reduced glutathione (GSH) and suppressed by the oxidative stress regulator GSK3-β. Our results demonstrate that GFPT2 controls growth and invasion in the D492 EMT model, is a marker for oxidative stress, and associated with poor prognosis in claudin-low breast cancer.

## Highlights

• GFPT2 is upregulated following EMT.
• GFPT2 is a marker for claudin-low breast cancer.
• GFPT2 affects vimentin, cell proliferation, and cell invasion.
• GFPT2 responds to oxidative stress.
• GFPT2 is regulated by insulin and EGF.

## Graphical Abstract

**Figure:**
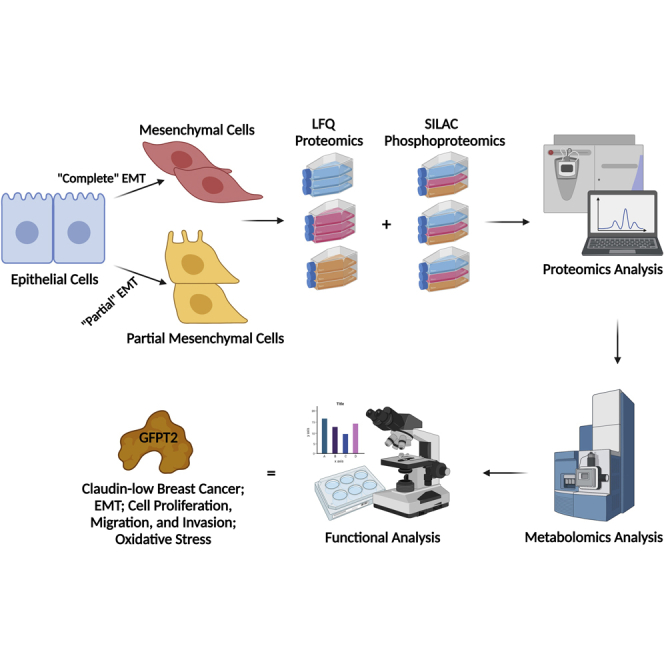

## In Brief

Epithelial–mesenchymal transition (EMT) is a cellular process inherent to cancer cell metastasis. Metabolic reprogramming is a driver of EMT. We performed proteomic profiling of three isogenic cell lines from human breast epithelium representing the epithelial, mesenchymal, and “partial” mesenchymal states of EMT to identify metabolic vulnerabilities associated with cell invasion. Bioinformatic and functional analysis revealed that the metabolic enzyme GFPT2 is a marker of claudin-low breast cancer, responds to oxidative stress, and impacts EMT, cell growth, and cell invasion.

Breast cancer is the most prevalent cancer in women worldwide (1). Within 3 years after the initial diagnosis, around 10 to 15% of patients with breast cancer develop distant metastasis (2). Epithelial–mesenchymal transition (EMT) is a natural process during embryonic development that tumor cells hijack to gain migration and invasive properties (3, 4). EMT is characterized by a broad spectrum of epithelial–mesenchymal states that ultimately affect cancer malignancy (5, 6).

Multiple changes to metabolism accompany breast cancer. These include changes to enzymes in glycolysis (7), the tricarboxylic acid (TCA) cycle (8, 9), and fatty acid synthesis (10). More recently, changes in serine and proline biosynthesis (8, 11) and nucleotide metabolism (12) have been described. However, definitive metabolic phenotypes that differentiate between noninvasive complete EMT and partial EMT with invasive potentials remain elusive (13). Understanding how regulation of enzyme activity on the protein level affects invasiveness may improve breast cancer personalized therapeutic interventions.

In this study, we set out to define changes in metabolic enzymes that accompany EMT in the EMT cell model D492 (14, 15) reviewed in Briem *et al.*, 2019 (16). The D492 breast EMT cell model contains three isogenic phenotypes: the epithelial D492 cells, the mesenchymal D492M cells, and the partial mesenchymal D492HER2 cells. D492 is a basal-like human breast epithelial cell line derived from normal tissue. The D492 cell line expresses both luminal (K8, K19) and myoepithelial (K5/6, K14) cytokeratins. It has epithelial stem cell properties and can differentiate into luminal and myoepithelial cells (15, 17). EMT in cultured breast epithelial cells can be triggered with growth factors such as TGF-β and EGF (14, 18, 19). It can also be induced *via* overexpression of certain EMT markers such as TWIST (20). D492M is a mesenchymal cell line spontaneously generated by 3D coculture of D492 with human endothelial cells in the absence of any dominant EMT inducers (15). Although the D492 and D492M cells are premalignant and not tumorigenic, the D492 cells gain tumorigenicity when HER2 is overexpressed. The D492HER2 cells have a partial mesenchymal phenotype, indicating that cells have gone through EMT (14). The D492 EMT cell model thus comprises three cell lines allowing different states of EMT to be studied *in vitro*. We hypothesized that comparative proteomics analysis of these three cell lines would highlight crucial metabolic enzymes to EMT in breast epithelium and discriminate metabolic enzymes that impart invasion properties.

We have previously defined changes to metabolism in the D492 EMT model within genome-scale metabolic network models. Glycan metabolism, amongst others, was altered in EMT (9). These models were based on changes to gene expression and extracellular metabolomic measurements. In this study, we analyzed the metabolic changes in EMT on the protein level, emphasizing mesenchymal cells that possess invasive potentials. We first positioned the D492 EMT cell model within the breast cancer cell model landscape based upon the LFQ and SILAC proteomics data. We then identified the hexosamine biosynthesis pathway (HBP) rate-limiting enzyme, glutamine-fructose-6-phosphate transaminase 2 (GFPT2), as a potential target in claudin-low breast cancer progression.

Enzymes involved in glycan processing were overrepresented in both mesenchymal proteomes, and the HBP rate-limiting enzyme GFPT2 was upregulated in D492M and further still in the D492HER2 mesenchymal cells as compared with D492. Metabolomics analysis confirmed changes to HBP flux. We then compared GFPT2 expression across clinical breast cancer subtypes and breast cancer cell lines and knocked down *GFPT2* to assess its effects on the EMT program, cell growth, and cell invasion in the D492 EMT model. These analyses suggest that GFPT2 is a tumor promoter in claudin-low breast cancer. The role of GFPT2 in mediating glycan synthesis has been reported in a series of studies that show that GFPT2 mediates response *via* glycosylation of master regulators of metabolism, including NF-κB and β-catenin (21, 22). The function of GFPT2 in glutaminolysis is less explored, although its importance has been inferred from its enzymatic activity. In light of recent results that show that altered glutaminolysis in the D492 EMT model influences their ability to synthesize glutathione from glutamine-derived glutamate, and that this influences their susceptibility to cancer therapeutics (23), we explored the role of GFPT2 in maintaining redox balance in EMT.

## Experimental Procedures

### Cell Culture

D492, D492M, D492HER2, and D492DEE were generated in-house (14, 15, 17) and cultured in serum-free H14 medium. The MDA-MB-231 cell line was cultured in RPMI 1640 (Thermo, 52400-025) supplemented with 10% Fetal Bovine Serum (FBS, Gibco 10270106) and 100 IU penicillin and 0.1 mg/ml streptomycin (Gibco, 15140122). Cells were at 37 °C and 5% CO_2_ for routine maintenance. The H14 medium is Dulbecco's modified Eagle's medium – F12 (DMEM/F12 without glutamine, Thermo, 21331020) supplemented with 250 ng/ml insulin (Merck, I6634), 10 μg/ml transferrin (Merck, T2252), 10 ng/ml EGF (PeproTech, AF-100-15), 2.6 ng/ml Na-selenite (BD Biosciences, 354201), 10^−10^ M estradiol (Sigma, E2758), 1.4 × 10^−6^ M hydrocortisone (Sigma, H0888), 0.15 IU prolactin (PeproTech, 100-07), 100 IU penicillin & 0.1 mg/ml streptomycin, and 2 mM glutamine (Thermo, 25030024). In the SILAC proteomic experiment, DMEM-F12 was replaced by “DMEM:F-12 for SILAC” (Thermo, 88370) with “light-,” “medium-,” or “heavy-labeled” arginine or lysine (Cambridge Isotope Laboratories). In the ^13^C labeling experiment, the base medium was changed to “DMEM, no glucose, no glutamine, no phenol red” (Thermo, A1443001), and ^13^C labeled 1,2-glucose, ^13^C labeled 1-glutamine, or ^13^C labeled 5-glutamine from Cambridge Isotope Laboratories was added. Medium excluded penicillin and streptomycin for the transient knockdown experiments according to instruction. In the invasion assay, H14 was supplemented with 10% FBS. Cell cultures were routinely checked for *mycoplasma* contamination.

### Label-free Quantification (LFQ) Proteomics

#### Protein and Peptide Sample Preparation

Cells were cultured in T75 flasks in triplicates (three flasks per cell line), and the seeding density was 600,000 cells per flask. Proteins were harvested at 90% confluency, and 72 h after seeding, cells were washed twice with ice-cold PBS and lysed by 450 μl lysis buffer containing 4% sodium dodecyl sulfate (SDS, MP Biomedicals) in 100 mM Tris (Sigma). Flasks were kept on ice for 10 min. The cell lysates were transferred to 1.5 ml Eppendorf tubes. After five freeze (−80 °C)/thaw (room temperature) cycles, the sample was spun at 20,718*g* for 20 min at 4 °C. The supernatant was collected and aliquoted in new tubes and stored at −80 °C. Protein quantification was measured with BCA protein assay (Pierce).

For Filter-Aided Sample Preparation (FASP), an equivalent of 300 μg of proteins in 150 μl from each sample was reduced with 100 mM dithiothreitol (DTT), and samples were then processed using FASP protocol (24). Proteins on the filters were digested twice at 30 °C with trypsin (enzyme-to-substrate ratio: 1:100 (w/w); 3 μg × 2), first overnight and then for another 6 h in a final volume of 200 μl. The resulting peptides were desalted using a C18 solid-phase extraction cartridge (Empore, Agilent technologies). Peptides were resuspended in 50 μl 1% formic acid and quantified using pierce quantitative colorimetric peptide assay (product 23275, Thermo Scientific).

#### LC-MS/MS Analysis

Trypsin-digested peptides were separated using an Ultimate 3000 RSLC (Thermo Scientific) nanoflow LC system. In total, 130 ng of peptides was loaded with a constant flow of 5 μl/min onto an Acclaim PepMap100 nanoViper C18 trap column (100 μm inner-diameter, length: 2 cm; Thermo Scientific). After trap enrichment, peptides were eluted onto an EASY-Spray PepMap RSLC nanoViper, C18, particle size: 2 μm, pore size: 100 Å column (75 μm inner-diameter, length: 50 cm; Thermo Scientific) with a linear gradient of 2 to 35% solvent B (80% acetonitrile with 0.08% formic acid, Solvent A – 0.1% formic acid) over 124 min with a constant flow of 300 nl/min and column temperature of 50 °C. The HPLC system was coupled to a linear ion trap Orbitrap hybrid mass spectrometer (LTQ-Orbitrap Velos, Thermo Scientific) *via* an EASY-Spray ion source (Thermo Scientific). The spray voltage was set to 1.8 kV, and the temperature of the heated capillary was set to 250 °C. Full-scan MS survey spectra (m/z 335–1800) in profile mode were acquired in the Orbitrap with a resolution of 60,000 after accumulation of 1,000,000 ions. The 15 most intense peptide ions from the preview scan in the Orbitrap were fragmented by collision-induced dissociation (CID, normalized collision energy, 35%; activation Q, 0.250; and activation time, 10 ms) in the LTQ after the accumulation of 5000 ions. Maximal filling times were 1000 ms for the full scans and 150 ms for the MS/MS scans. Precursor ion charge state screening was enabled, and all unassigned charge states, as well as singly charged species, were rejected. The lock mass option was enabled for survey scans to improve mass accuracy (25). Data were acquired using the Xcalibur software.

#### Peptide and Protein Identification and Quantification

The raw mass spectrometric data files were collated into a single quantitated dataset using MaxQuant (version 1.5.2.8) (26) and the Andromeda search engine software (27). Enzyme specificity was set to that of trypsin, allowing for cleavage N-terminal to proline residues and between aspartic acid and proline residues. Other parameters used were: (i) variable modifications—methionine oxidation, protein N-acetylation, gln → pyro-glu, phospho (STY), deamidation (NQ); (ii) fixed modifications, cysteine carbamidomethylation; (iii) database: Uniprot-human-up5640 (release date of sequence database searched: 05.2017; number of entries: 20,201); (iv) LFQ: min ratio count, 2 (v) MS/MS tolerance: FTMS- 10 ppm, ITMS- 0.6 Da; (vi) maximum peptide length, 6; (vii) maximum missed cleavages, 2; (viii) maximum labeled amino acids, 3; and (ix) false discovery rate (FDR), 1%. LFQ intensities were reported individually for each sample and were given as a relative protein quantitation across all samples. LFQ intensities were represented by a normalized intensity profile as described by Cox (28) affording a matrix with number of samples and number of protein groups as dimensions. The iBAQ quantification was carried out in MaxQuant (version 1.5.2.8) for the same raw data obtained. The same parameters as described above for the LFQ quantification were applied for the iBAQ quantification except for the selection of the iBAQ method for outputs.

Protein identification was defined as one or more identified peptides observed in at least two out of three replicates in at least one cell line. Protein quantification was calculated when at least two out of three replicates in at least one cell line had detectable intensities.

### Stable Isotope Labeling by Amino Acids in Cell Culture (SILAC) (Phospho)Proteomics

#### Protein and Peptide Sample Preparation, Fractionation, and Enrichment

##### Protein Extraction

The cell lines D492M, D492, and D492HER2 were labeled with “light,” “medium,” and “heavy” stable isotope-labeled versions of arginine and lysine for SILAC analysis, respectively. The SILAC labeling was not randomized among the cell lines for the triplicates. Cells were first cultured in T25 flasks with respective SILAC labels to get fully labeled cell populations for D492 (“medium” label, L-arginine-^13^C_6_ hydrochloride (Arg +6 Da), L-lysine-4,4,5,5-d4 hydrochloride (Lys +4 Da)), D492M (“light” label, L-arginine, L-lysine), and D492HER2 (“heavy” label, L-arginine-^13^C_6_,^15^N_4_ hydrochloride (Arg +10 Da), L-lysine-^13^C_6_,^15^N_2_ hydrochloride (Lys +8 Da)). The D492 and D492M cells were cultured in the “medium-” and “light-labeled” medium for six passages to ensure that the cells were close to the fully labeled status. The D492HER2 cells were cultured in the “heavy-labeled” medium for five passages. To harvest enough proteins, cells were propagated in T75 flasks (Santa cruz), then cultured in T182 flasks (Santa cruz) in triplicates, and the seeding density was 1,500,000 cells per flask, which was calculated to be consistent with the LFQ proteomics experiment. The same procedures as described in the LFQ protein preparation section were conducted for SILAC protein extraction with lysis buffer supplemented with one tablet of PhosSTOP phosphatase inhibitors (Roche) and one tablet of cOmplete mini EDTA-free protease inhibitors (Roche).

##### Protein Digestion (FASP Processing of Samples)

Proteins were solubilized in 150 μl of Tris-HCl (100 mM, pH 7.6) containing 4% SDS and 100 mM DTT. Protein extracts were heated at 95 °C, and DNA was shredded by sonication to reduce the viscosity of the lysates. Samples were then centrifuged and processed using FASP protocol (24) with some modifications. After lysates were passed through the filters (Nanosep, 10k, PALL Life Sciences), proteins were alkylated in 100 μl iodoacetamide (IAA) at a final concentration of 50 mM for 15 min, filters were washed four times with 200 μl 8 M urea in Tris-HCl (100 mM, pH 8), then twice with 200 μl 40 mM ammonium bicarbonate. Proteins on the filters were then digested twice at 30 °C with trypsin (enzyme-to-substrate ratio: 1:100 (w/w); 3.3 μg × 2), first overnight and then for another 6 h in 200 μl, ammonium bicarbonate at 40 mM. The resulting tryptic peptides were desalted using a C18 solid-phase extraction cartridge (Empore, Agilent technologies).

##### Peptide Fractionation (High pH Reverse-phase Fractionation)

Samples equivalent to 4 mg were dissolved in 200 μl of 10 mM ammonium formate buffer (pH 9.5), and peptides were fractionated using high pH RP chromatography. A C18 column from Waters (XBridge peptide BEH, pore size: 130 Å, particle size: 3.5 μm, inner-diameter: 4.6 × length: 150 mm, Ireland) with a guard column (XBridge, C18, particle size: 3.5 μm, inner-diameter: 4.6 × length: 20 mm, Waters) was used on an Ultimate 3000 HPLC (Thermo-Scientific). Buffers A and B used for fractionation consisted, respectively of 10 mM ammonium formate in distilled, deionized water (Buffer A) and 10 mM ammonium formate in 90% acetonitrile (Buffer B), and both buffers were adjusted to pH 9.5 with ammonia. Fractions were collected using a WPS-3000FC autosampler (Thermo-Scientific) at 1 min intervals. Column and guard column were equilibrated with 2% buffer B for 20 min at a constant flow rate of 0.75 ml/min and a constant temperature of 21 °C. Samples (185 μl) were loaded onto the column at 0.75 ml/min, and the separation gradient started from 2% buffer B to 5% B in 6 min, then from 5% B to 60% B within 55 min. The column was washed for 7 min at 100% buffer B and equilibrated at 2% buffer B for 20 min, as mentioned above. The fraction collection started 1 min after injection and stopped after 80 min (total of 80 fractions, 750 μl each). Each peptide fraction was acidified immediately after elution from the column by adding 20 to 30 μl 10% formic acid to each tube in the autosampler. The total number of fractions concatenated was set to 10, with 96% of material from each fraction was used for phospho-enrichment, and 4% was used for total proteome analysis. The content of the fraction from each set was dried prior to further analysis.

##### Phosphoproteomic Phospho-peptide Enrichment

Phospho-peptide enrichment was performed using MagReSyn-TiIMAC beads (Resyn Biosciences) and Magnetic Rack (DynaMag-2, Life Technologies). Tryptic peptides to TiIMAC beads were used at 1:5 ratio (w/w). Beads were first washed using Magnetic Rack with 80 μl, 1% NH_4_OH or ammonia, followed with 200 μl acetonitrile. TiIMAC beads were equilibrated for 2 min with gentle mixing in 200 μl loading buffer consisting of 1 M glycolytic acid 80% acetonitrile and 5% trifluoroacetic acid (TFA). Dried samples were resuspended in 100 μl loading buffer, added to TiIMAC beads, and the mixture was incubated with gentle mixing for 20 min at room temperature (RT). Samples were then washed for 2 min successively with 200 μl loading buffer, three times with 200 μl of 80% acetonitrile-1% TFA, and finally with 200 μl of 10% acetonitrile-0.2% TFA. Phospho-peptides were eluted from beads three times using 80 μl of 1% ammonia, and gentle mixing with pH immediately lowered to 2 using 10% formic acid. Eluted phospo-peptides were pooled, dried in speed vac at RT, and stored at −80 °C before LC-MS analysis.

#### LC-MS/MS Analysis

Analysis of peptides for total proteome and phospho-proteome was performed on a Velos-Pro orbitrap (Thermo Scientific) mass spectrometer coupled with a Dionex Ultimate 3000 RS (Thermo Scientific). LC buffers were the following: buffer A (2% acetonitrile and 0.1% formic acid in distilled, deionized water (v/v)) and buffer B (80% acetonitrile and 0.08% formic acid in distilled, deionized water (v/v). All fractions from both total proteome and phospho-proteome were reconstituted in 50 μl of 1% formic acid. An aliquot (10 μl of total proteome; 15 μl of phospho-proteome) of each fraction was loaded at 10 μl/min onto a trap column (inner diameter: 100 μm × length: 2 cm, PepMap nanoViper C18 column, particle size: 5 μm, pore size: 100 Å, Thermo Scientific) equilibrated in buffer A for 19 min. The trap column was washed for 6 min at the same flow rate, and then the trap column was switched in-line with a Thermo Scientific, resolving C18 column (inner diameter: 75 μm × length: 50 cm, PepMap RSLC C18 column, particle size: 2 μm, pore size: 100 Å) kept at a constant temperature of 50 °C. Peptides were eluted from the column at a constant flow rate of 300 nl/min with a linear gradient from 5% buffer B to 35% buffer B within 124 min. The column was then washed for 20 min at 98% buffer B and re-equilibrated in 5% buffer B for 19 min. LTQ-Orbitap Velos Pro was operated in data-dependent positive ionization mode (DDA). The source voltage was set to 2.6 Kv, and the capillary temperature was 250 °C.

A scan cycle comprised MS1 scan (m/z range from 335 to 1800) in the velos pro-orbitrap followed by 15 sequential dependent MS2 scans (the threshold value was set at 5000, and the minimum injection time was set at 200 ms) in LTQ with CID. The resolution of the Orbitrap Velos was set at 60,000 after the accumulation of 1,000,000 ions. Precursor ion charge state screening was enabled, with all unassigned charge states and singly charged species rejected. Multistage activation for neutral loss ions was activated only for analysis of phospho-peptides. The lock mass option was enabled for survey scans to improve mass accuracy. To ensure mass accuracy, the mass spectrometer was calibrated on the first day that the runs were performed.

#### Peptide and Protein Identification and Quantification

The MaxQuant setup and parameters for SILAC were consistent with the LFQ experiment described in the previous section with several differences: (i) variable modifications, methionine oxidation, protein N-acetylation, gln → pyro-glu, Phospho (STY); (ii) database: Uniprot-human_dec2017 (release date of sequence database searched: 12.2017; number of entries: 20,244); (iii) “heavy” label: R10K8, “medium” label: R6K4. Peptide ratios were calculated for each arginine- and/or lysine-containing peptide as the peak area of labeled arginine/lysine divided by the peak area of nonlabeled arginine/lysine for each single-scan mass spectrum. Peptide ratios for all arginine- and lysine-containing peptides sequenced for each protein were averaged. Data were normalized using 1/median ratio value for each identified protein group per labeled sample. Phospho-peptides were normalized using the nonphospho protein 1/median values to correct for mixing errors and compared against the individual nonphospho protein ratio itself to correct for protein regulation interactions. Different parameters used in the iBAQ quantification were: (i) variable modifications—methionine oxidation, protein N-acetylation, Phospho (STY), deamidation (NQ); (ii) database: Homo_sapiens.GRCh38.pep.all (release date of sequence database searched: 06.2018; number of entries: 107,844); (iii) MS/MS tolerance: FTMS- 20 ppm, ITMS- 0.5 Da.

Valid SILAC quantification was defined as when two out of three replicates were generated with valid SILAC ratios. Valid phosphoproteomic quantification was filtered by localization probability >0.75 in all three replicates.

### Transient Knockdown With siRNA and Quantitative Reverse Transcription PCR (RT-qPCR)

Cells were seeded either at 60,000 cells/well in 48-well plates or at 480,000 cells/well in 6-well plates. Prior to cell seeding, plates were coated with respective control siRNA (Silencer Select Negative Control, 4390843), GFPT2 target siRNAs (Silencer Select siGFPT2, s19305 and s19306), GSK3B target siRNAs (Silencer Select siGSK3B, s6239 and s6241), and RELA target siRNAs (Silencer Select siRELA, s11914 and s11915) as well as Lipofectamine RNAiMAX Transfection Reagent (Thermo). Cells were transfected at 37 °C and 5% CO_2_ for 48 h with a final siRNA concentration of 10 nM.

In the RT-qPCR experiments, cells were mainly cultured in 48-well plates for 72 h, followed by total RNA extraction with TRI Reagent Solution (Invitrogen). RNA concentration was determined in NanoDrop One (Thermo). In total, 1000 ng of RNA was used for cDNA synthesis on the thermal cycler (MJ research, PTC-225, Peltier Thermal Cycler) using High-Capacity cDNA Reverse Transcription Kit (Thermo). Gene expression was measured with SYBR Green (Luna Universal qPCR Master Mix, NEW ENGLAND BioLabs) on Bio-Rad CFX384 Touch Real-Time PCR Detection System (Bio-Rad). Primers were selected either based on literature or from PrimerBank or designed on Primer3Plus website. Primer sequences for genes in this study (TAG Copenhagen) were listed in supplemental Table S1. The VIM primers were from IDT (Hs.PT.58.38906895).

### Western Blot

Cells were incubated with siRNAs as described above. Protein lysates were extracted with RIPA buffer (Pierce, 89900, Thermo) supplemented with protease and phosphatase inhibitors (Halt, 1861284, Thermo) and quantified with BCA protein assay. Proteins were separated on 4 to 12% Bis-Tris gels (NuPAGE, Thermo), transferred to polyvinylidene fluoride (PVDF) membranes (IPFL00010, Immobilon), and probed with antibodies against O-GlcNAcylation (1:200 dilution; sc-59623; Santa Cruz Biotechnology) and the loading control, β-actin (1:2000; MA5-15739; Thermo). The Western blot detection reagents were Clarity Max Western ECL substrate (Bio-Rad), and plots were imaged in the Molecular Imager ChemiDoc XRS+ Systems (Bio-Rad).

### Proliferation, Scratch, and Invasion Assay

#### Proliferation Assay

Cells in quadruplicates were seeded at 10,000 cells/well in 96-well plates. *GFPT2* knockdown followed the methods described above. For D492 and D492M, 24 h after seeding (48 h for D492HER2), cells were placed under the microscope (LEICA CTR 6500, bright field, 10×) with 5% CO_2_ at 37 °C for real-time monitoring and multiple data acquisition. This was controlled by software Micro-Manager 1.4.22. Three spots were chosen in each well, and photos were taken every 6 h. Cell growth was monitored for 66 h for D492 and D492M while 42 h for D492HER2. Photos were batch-processed with Macro in software ImageJ 1.52p, and cell numbers were normalized to the starting time point under the microscope.

#### Scratch Assay

The scratch assay was performed in the IncuCyte ZOOM system (2018A) following the manufacturer’s instructions. Cells in triplicates were seeded at 40,000 cells/well in 96-well plates (Essen bioscience, ImageLock, 4379). *GFPT2* knockdown followed the methods described above. Cells were scratched and put into the IncuCyte after 48 h of transfection with siRNAs. The IncuCyte ZOOM system took pictures every 2 h. Two positions in each well were chosen, and cells were monitored for 72 h to reach full wound closure. Images were analyzed in the software IncuCyte ZOOM (2018A), and wound confluence data were exported.

#### Invasion Assay

The D492HER2 cells were cultured with siRNA transfection (Scramble and siGFPT2) for 48 h in a 6-well plate. *GFPT2* knockdown followed the methods described above. Cells were then reseeded into filter units (Falcon Permeable Support for 24-well Plate with 8.0 μm Transparent PET Membrane, 353097) coated with Matrigel (Corning Matrigel Matrix, 356234) at a density of 30,000 cells/well. First, the filter inserts were coated with 100 μl 1:10 diluted Matrigel for 20 to 30 min at 37 °C. Next, 300 μl of cell suspension was added on top of the filter units. Then, 500 μl of H14 medium with 10% FBS was added to the wells in the 24-well plates below the filters. Finally, cells were incubated at 37 °C and 5% CO_2_ for 48 h. Noninvasive cells on top of the filters were removed with cotton swabs, followed by fixation with paraformaldehyde (PFA, 3.7%, Sigma, 252549) and DAPI staining (1:5000, Sigma, D9542). Ten images per filter unit were taken by the EVOS FL Auto Imaging System (10×, Thermo), followed by the batch analysis of the images in Macro ImageJ 1.52p. To normalize the different cell numbers in the filter units, cells were seeded into a 24-well plate along with the filter units, and they were cultured and treated in the same way as cells in the filter units.

### Metabolomics Analysis

In the *GFPT2* knockdown experiments, cells in triplicates were transfected with control siRNA (scramble), target siRNA (siGFPT2), or neither (wide-type cells) for 48 h in 6-well plates, then cultured for another 24 h before metabolite extraction. In ^13^C labeling experiments, wide-type cells were cultured in T25 flasks in triplicates, and after cells reached 80% confluency, the medium was changed to ones without glucose or glutamine. After culturing cells in the medium without glucose or glutamine for 4 h (as Time 0), labeled ^13^C 1,2-glucose or ^13^C 1(5)-glutamine was added. Metabolites were extracted at time 0 and after 6 h. Metabolites were extracted with cold 80% MeOH supplemented with metabolite internal standards as instructed in an in-house protocol. Extracts were analyzed on the UPLC mass spectrometry (SYNAPT G2, Waters) according to published protocols (29). For ^13^C labeling experiments, data were analyzed in ISOCORE, and we normalized the mean enrichment of ^13^C in UDP-N-acetylglucosamine (UDP-GlcNAc) to the total amount of UDP-GlcNAc and presented it as relative ^13^C incorporation. Metabolomic data were normalized to protein levels.

### Hydrogen Peroxide (H_2_O_2_) and Reduced Glutathione (GSH) Treatment and Growth Factor Deprivation

The MDA-MB-231 cells were seeded in 24-well plates at 300,000 cells/well and cultured for 48 h followed by treatment with 2 μM hydrogen peroxide (H_2_O_2_, Honeywell, 18304H) for 2 h. GFPT2 gene expression was tested by RT-qPCR.

The MDA-MB-231 cells were seeded in 24-well plates at 200,000 cells/well and cultured for 24 h, followed by treatment with 50 mg/l reduced glutathione (GSH, Sigma, G4251) for 48 h. Cells were changed with fresh GSH medium 2 h before the RNA extraction. GFPT2 gene expression was tested by RT-qPCR.

The MDA-MB-231 cells were cultured in the H14 medium as described for the D492 cell lines, then seeded in 24-well plates at 200,000 cells/well and cultured for 24 h followed by treatments with medium deprived of insulin or EGF for 48 h. Fresh medium was changed for the cells 2 h before the RNA extraction. GFPT2 gene expression was tested by RT-qPCR.

### Glutathione Assay

The glutathione levels, including both reduced (GSH) and oxidized (GSSG) glutathione, were measured with the GSH/GSSG-Glo Assay from Promega (V6611). Cells in quadruplicates were seeded at 20,000 cells/well in 96-well plates. *GFPT2* knockdown and H_2_O_2_ treatments followed the methods described above. The glutathione levels were measured 24 h after changed medium. The luminescence signal was detected in the microplate reader (SpectraMax M3, Molecular Devices) with white and opaque 96-well plates (BRANDplates, 781965). To normalize the glutathione level, cells were counted using a crystal violet assay. In short, cells were fixed with 100% cold MeOH and stained with 0.25% crystal violet (Merck, C.I. 42555). After washing, stained cells were dissolved into 100 μl of 10% acetic acid and measured at 570 nm in the microplate reader (SpectraMax M3, Molecular Devices LLC).

### Experimental Design and Statistical Rationale

We conducted two types of proteomics analysis (single-shot LFQ and SILAC with ten fractions) to increase the validity and reproducibility of our results. In the LFQ experiment were four different cell lines (D492, D492M, D492HER2, and D492DEE) in three biological replicates with 12 samples analyzed and described to yield statistical significances. In the SILAC experiment were three different cell lines (D492, D492M, and D492HER2) in three biological replicates with nine samples analyzed and described due to the maximum labeling capacity in SILAC. In both LFQ and SILAC, the epithelial D492 cells were used as controls for the two mesenchymal cell lines. Statistical analysis for all the comparisons between different treatments was conducted in R (two-sided one or two sample(s) Student’s *t* test) for the (phospho)proteomic data, metabolomic data, and functional analyses. All error bars represent standard deviation (SD).

The heatmaps and dendrogram were generated in R with packages “ComplexHeatmap,” “ggdendro,” and “dendextend” (30, 31). Volcano plots were plotted in R with data analyzed in Perseus (version 1.6.2.3, Replace missing values from normal distribution, two-sided Student’s *t* test for LFQ, one sample *t* test for SILAC, Permutation-based FDR). GO annotation was performed in Perseus (version 1.6.12.0, Fisher exact test, Benjamini–Hochberg FDR) (32) using all identified proteins from the SILAC experiment as background. We used the R package “pathfindR” (100 iterations; Protein–protein interaction: Biogrid; *p*-values adjustment: “bonferroni,” adjusted *p*-value threshold: 0.05) (33) to enrich KEGG pathways. Reactome metabolic pathways were enriched with the default parameters on the Reactome website (Version 65, 67, and 72 were used for D492 *versus* D492M, D492 *versus* D492HER2, and D492M *versus* D492HER2, respectively) (34) and plotted as treemaps in R. The protein interaction networks of proteins involved in the metabolic pathways (enrichment FDR < 0.05) were created in STRING (Version 11.0; k-means clustering, minimum required interaction scores: medium confidence 0.400) (35) and visualized in Cytoscape (version 3.5.1/Version 3.6.1) (36). Survival analysis in breast cancer patients was performed in R with packages: “survminer” and “survival”. The top and bottom 20th percentile of patients were included in the analysis. Breast cancer patients’ data were acquired *via* the Cancer Genome Atlas (TCGA) cBioPortal (Breast Invasive Carcinoma (TCGA, Provisional)) (37). EMT markers were referenced to and downloaded from the online EMT database (38). RNA expression data of GFPT2 in breast cell lines and breast cancer patients had referred to the Cancer Cell Line Encyclopedia (CCLE) database (39), the Harvard Medical School (HMS) LINCS database (40), and TCGA cBioPortal (Breast Cancer (METABRIC, Nature 2012 & Nat Commun 2016)) (41), respectively. The scatter plots were plotted in R for proteins identified and quantified in both LFQ and SILAC (Pearson). Pathway enrichment of the phosphoproteomic data was performed by Ingenuity Pathway Analysis (IPA) (QIAGEN, version from 2018), while motif enrichment was done in the software Perseus. The R codes for figure plotting can be found on https://github.com/QiongW56/GFPT2_Publication_2021.

## Results

### The D492 and D492M Cell Lines Have Basal-like Proteomic Fingerprints, While D492HER2, Closer to D492M, Is Classified as Claudin-low

**Fig. 1:**
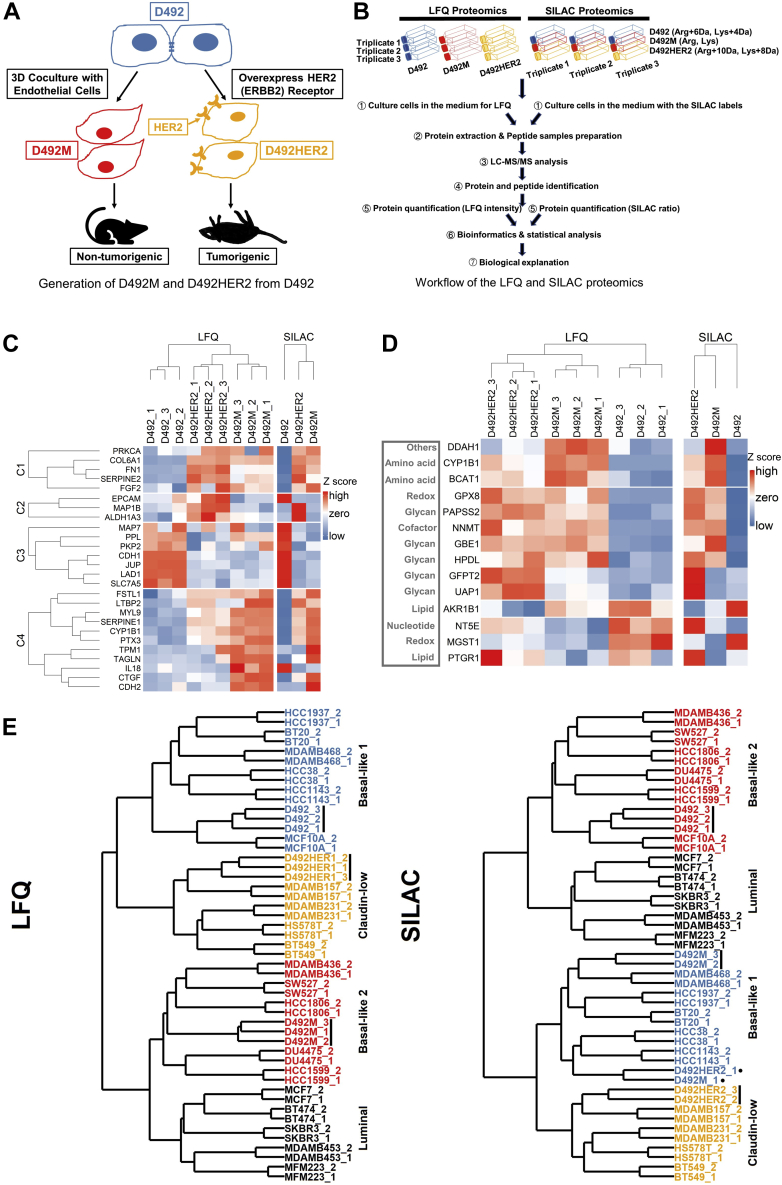
**Overview of the study and the D492 EMT cell model.***A*, the process of generating D492M and D492HER2 from D492. The D492 cells were cocultured with endothelial cells (BRENCs or HUVECs) to generate spindle colonies that were subcultured to generate a new cell line D492M. The D492M cells are nontumorigenic. The HER2 (ERBB) receptor was overexpressed on the D492 cells to generate the D492HER2 cell line, and these cells can form tumors in mice. *B*, an overview of the whole proteomic experimental setup in this study from cell culture of D492, D492M, and D492HER2 to the bioinformatic and biological analysis of the LFQ and SILAC proteomic datasets. *C*, dysregulation of EMT markers in independent and published gene expression studies (GES) of EMT, which focused on different cell types and treatment modalities (42). *D*, dysregulation of EMT metabolic makers in the D492 cell model compared with the literature. There was a consistency between LFQ (*left*) and SILAC (*right*) except for NT5E. SILAC was consistent with the literature. HPDL, AKR1B1, and MGST1 were in an opposite trend compared with the literature. The mesenchymal metabolic signature (MMS) in the literature (44) was referred to in this analysis. For detailed descriptions of each EMT marker mentioned in Figure 1, *C* and *D*, please refer to the supplemental Data 4. *E*, classification of the D492 cell model. Using the iBAQ expression of proteins identified in both literature and this study, D492, D492M, and D492HER2 were clustered with other preclassified breast cell lines (45). LFQ (*left*) classified D492 as “Basal-like 1” (in *blue*), D492M as “Basal-like 2” (in *red*), and D492HER2 as “Mesenchymal-like/claudin-low” (in *orange*), while SILAC (*right*) classified D492 as “Basal-like 2” (in *red*), D492M as “Basal-like 1” (in *blue*), and D492HER2 also as “Mesenchymal-like/claudin-low” (in *orange*). The LFQ and SILAC raw data were quantified by the iBAQ quantification method in MaxQuant.

The proteomes of the D492, D492M, and D492HER2 cell lines were investigated in biological triplicates by single-shot LFQ and SILAC proteomics with ten fractions (Fig. 1, *A* and *B* and supplemental Table S2). In the LFQ experiment (supplemental Data 1), we identified 28,766 peptides corresponded to 3595 protein groups (FDR < 1%). An increase of identified peptides (on average 68,692) was observed in the SILAC experiment due to the fractionation process (supplemental Data 2 and supplemental Fig. S1, *A*–*C*). The increased number of peptides in SILAC led to almost twofold more identified (FDR < 1%) and quantified proteins (5120 proteins) compared with that in the LFQ experiment (2705 proteins). The Pearson correlation between LFQ and SILAC was 0.685, 0.782, and 0.847 for ratios of D492M and D492HER2, ratios of D492HER2 and D492, and ratios of D492M and D492, respectively (supplemental Fig. S1, *D*–*F*). Cluster analysis showed more similarity between the proteomes of D492M and D492HER2 than to that of D492 (supplemental Fig. S1, *G* and *H* and supplemental Data 3). Furthermore, a comparison of unique proteins in D492 *versus* D492M revealed that the coverage of the proteome was altered by approximately 6.8% to switch between the two cellular phenotypes. For D492 *versus* D492HER2, this number was approximately 7.0%, and for D492M *versus* D492HER2, it was about 5.1% (supplemental Table S2).

The proteomic fingerprints of the D492, D492M, and D492HER2 cells were compared with the EMT gene expression signatures reported by Groger *et al.*, 2012 (42) (Fig. 1*C*). Twenty-six of the 130 reported EMT markers (42) were identified in both the LFQ and SILAC datasets. All but ALDH1A3 were consistent between this study and the literature (supplemental Data 4). D492M clustered with D492HER2, although clear differences were observed in the selected markers between D492M and D492HER2 (Fig. 1*C*). These proteins represented EMT markers whose expression was inconsistent in the tumorigenic *versus* nontumorigenic mesenchymal-like cell models. Particularly, genes in clusters C2 and C4 showed different trends in the two mesenchymal cell types. D492HER2 and D492M possessed different mesenchymal characteristics confirmed by the dbEMT2 database (43) (supplemental Fig. S2 and supplemental Data 5). Similar results were obtained when the datasets were compared with the mesenchymal metabolic signatures reported in Shaul *et al.*, 2014 (44) (Fig. 1*D* and supplemental Data 4).

To position the D492 EMT model in relation to other cell models of breast epithelium, we compared the D492, D492M, and D492HER2 proteomes with the fingerprints of breast cancer reported by Lawrence *et al.*, 2015 (45) (supplemental Data 6 and Fig. 1*E*). Both the SILAC and LFQ data placed D492 and D492M with basal-like breast cell lines while D492HER2 clustered with “mesenchymal-like/claudin-low” cell lines.

### Changes to Nucleoside Metabolism Accompany Nontumorigenic and Tumorigenic Mesenchymal Phenotypes in D492

**Fig. 2:**
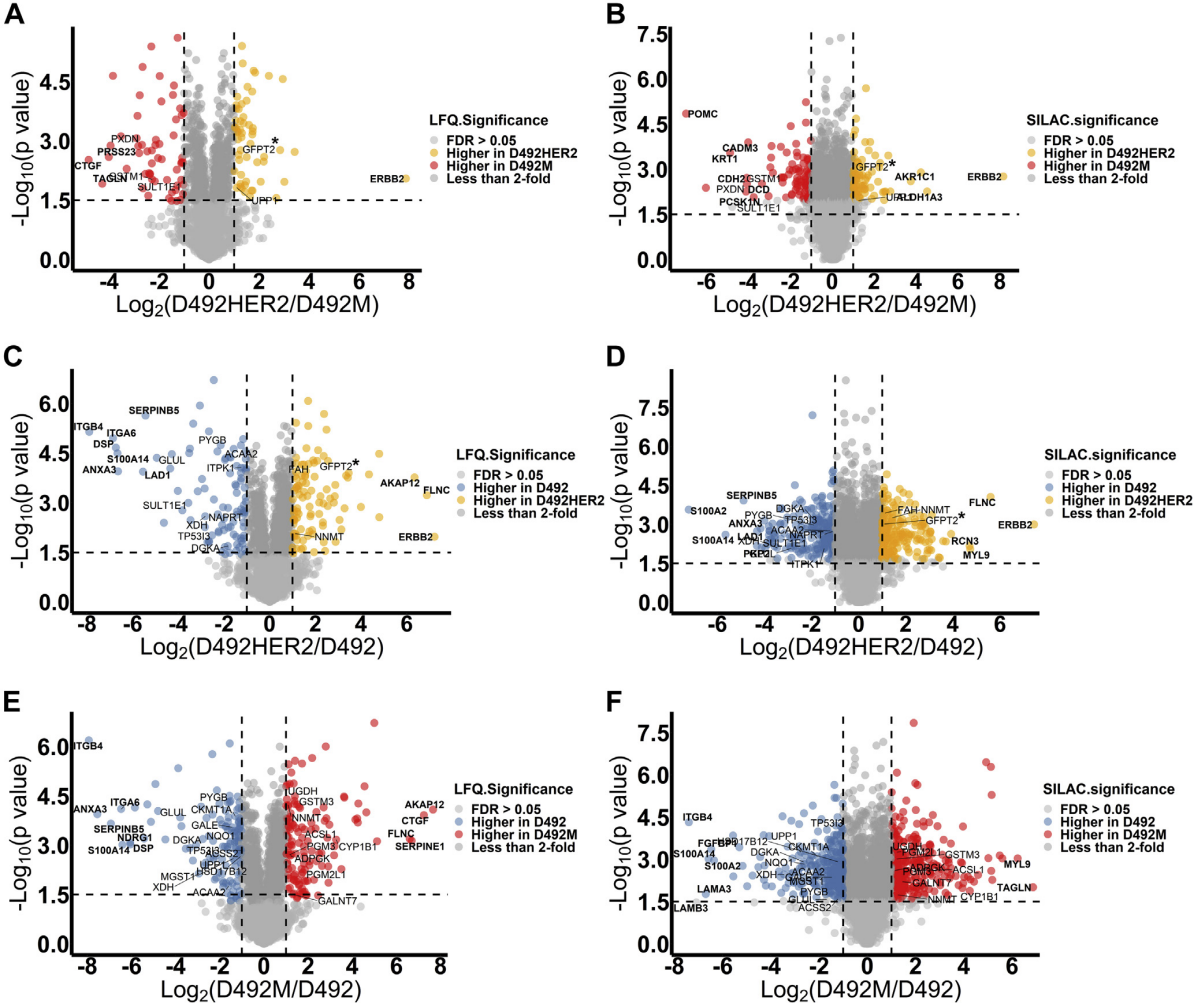
**LFQ and SILAC proteomic data plotting.** Statistical analysis of the LFQ and SILAC expressions of proteome in two different cell lines: D492HER2 *versus* D492M (*A* and *B*), D492HER2 *versus* D492 (*C* and *D*), and D492M *versus* D492 (*E* and *F*). Proteins with FDR less than 0.05 and fold change of more than 2 were colored. Metabolic enzymes differently expressed in two cell lines and consistent between LFQ and SILAC were labeled in the plots, and proteins with significant differences and big fold changes (at least fourfold between D492HER2 and D492M, fourfold between D492HER2 and D492, and sixfold difference between D492M and D492) were marked in *bold*. *Horizontal dash line* indicated −Log_10_(*p* value) at 1.5, and *vertical dash lines* indicated fold change at twofold. Proteins with the biggest differences between D492HER2 and D492M were PRSS23, CTGF, TAGLN, POMC, CADM3, KRT1, CDH2, DCD, PCSK1N, AKR1C1, ALDH1A3, and ERBB2, involved in cell adhesion and metabolism. Proteins differently expressed in D492HER2 and D492 were AKAP12, FLNC, ERBB, RCN3, MYL9, SERPINB5, ITGB4, ITGA6, DSP, S100A14, S100A2, LAD1, ANXA3, and PKP2, which were mainly involved in cell adhesion, structure, cell–cell interaction, and signaling. Lastly, a group of proteins that were similar to the differences observed with the other cell lines were differently expressed between D492M and D492, including AKAP12, CTGF, FLNC, SERPINE1, MYL9, TAGLN, ITGB4, ITGA6, ANXA3, SERPINB5, NDRG1, DSP, S100A14, FGFBP1, S100A2, LAMA3, and LAMB3. The main target in this study GFPT2 was highlighted with “∗” in the plots (*A*–*D*).

To identify specific proteins different between spontaneous nontumorigenic and the HER2-induced tumorigenic mesenchymal states, protein ratios and *p* values calculated for proteins in both the LFQ (Student's *t* test, two-sample tests, Permutation-based FDR < 0.05) and SILAC (Student's *t* test, one-sample tests, *p* value < 0.05) experiments were plotted for comparison (Fig. 2). Significantly deregulated proteins between the D492 epithelial phenotype and the two mesenchymal phenotypes shared in both the LFQ and SILAC datasets (supplemental Data 7) were analyzed by enrichment analysis of GO terms within Perseus (32) (supplemental Fig. S3, *A*–*C*). Of the identified GO terms, 11 were metabolic processes in HER2-induced tumorigenic EMT, while five metabolic processes were enriched in nontumorigenic EMT. Nucleotide-sugar metabolic process was enriched in both comparisons and was also different between the two mesenchymal cell lines. KEGG pathway analysis was also performed to complement these findings. Pathways involved in cell structure, migration, adhesion, invasion, and proteoglycans were enriched in the mesenchymal phenotypes compared with the epithelial phenotype. Nucleoside metabolism altered specifically between the two mesenchymal cell lines (supplemental Fig. S3, *D*–*F* and supplemental Data 8).

**Fig. 3:**
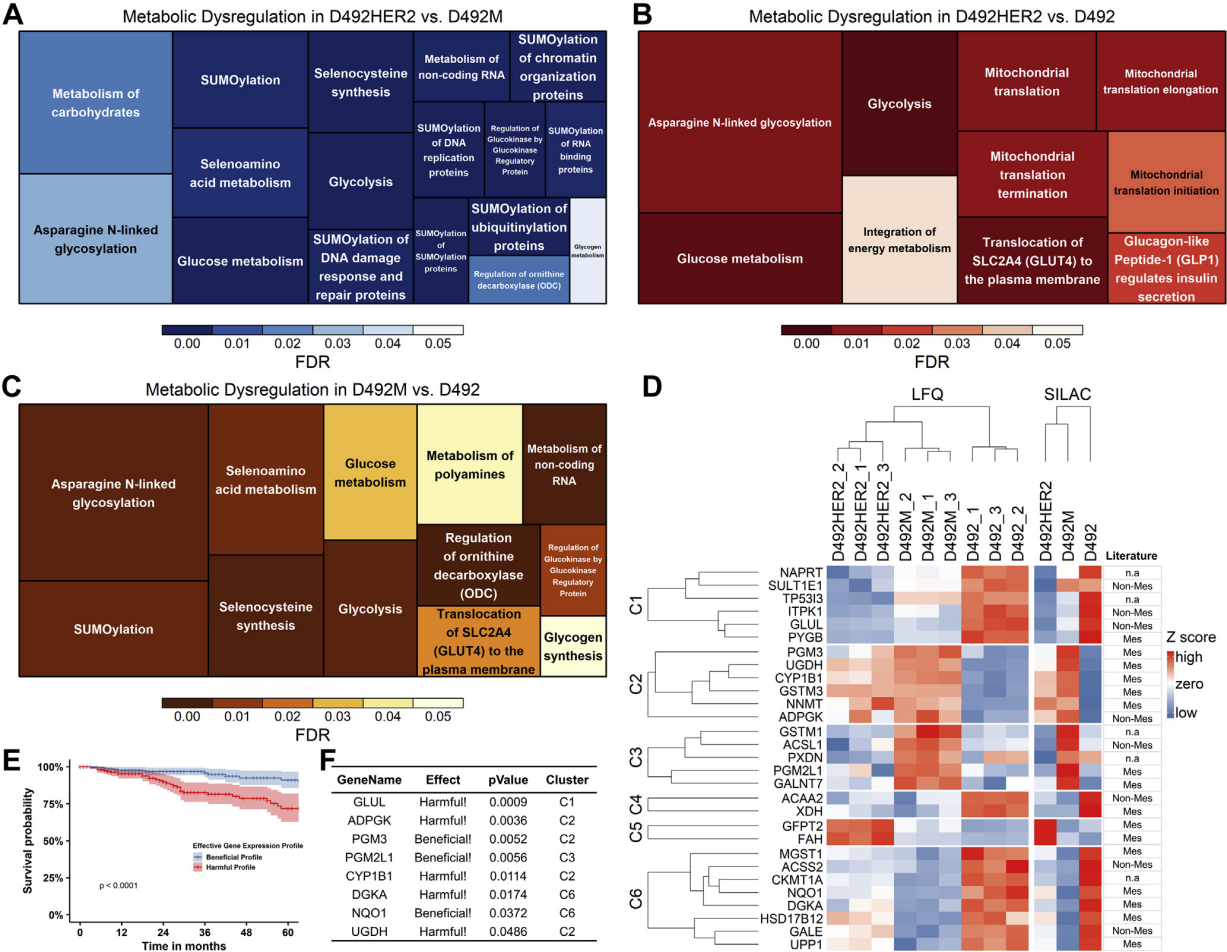
**Dysregulated metabolic pathways in two mesenchymal transitions and the metabolic targets identified in this study.***A-C,* Reactome metabolic pathways were differently enriched in D492HER2 *versus* D492M (*A*), D492HER2 *versus* D492, (*B*) and D492M *versus* D492 (*C*). Proteins involved in each Reactome metabolic pathway were plotted in supplemetal Fig. S4. (Data used for analysis: Supplemental Data 7; Student *t* test, Permutation-based FDR < 0.05, one sample *t* test, *p* value of SILAC ratio < 0.05). *D*, differentially expressed metabolic proteins in D492HER2 *versus* D492M, D492HER2 *versus* D492, and D492M *versus* D492 (student *t* test, permutation-based FDR <0.05 for LFQ, one sample *t* test, *p* value of SILAC <0.05, more than twofold in both LFQ and SILAC) were manually identified. Samples were in triplicates for both D492, D492M, and D492HER2 in the LFQ and SILAC experiments. For SILAC, the median relative expression of each target for D492, D492M, and D492HER2 was plotted. Targets were clustered into six clusters. The relative expression from the lowest to the highest for each metabolic protein was indicated in color scaling from *blue* to *red*, as shown in the color bar. On the *right side* was listed the identified metabolic targets in mesenchymal (Mes) and nonmesenchymal (Non-Mes) groups based on literature (44). n.a: not available in literature. *E* and *F*, survival analysis of the identified metabolic targets in breast cancer patients of all types revealed a group of enzymes might affect the outcome of patients’ survival. The enzymes that exerted either beneficial or harmful effects on patients were listed in the table (*F*) together with the *p* value for each enzyme’s effect on patients. Clusters in which the enzymes resided were listed too. TCGA data, Breast Invasive Carcinoma (TCGA, Provisional), were used in this analysis.

Focusing on metabolism, we mapped 102 (D492HER2 *versus* D492M), 84 (D492HER2 *versus* D492), and 119 (D492 *versus* D492M) differentially expressed metabolism-related proteins to their respective metabolic pathways (Fig. 3, *A*–*C* and supplemental Fig. S4). Asparagine N-linked glycosylation, glycolysis, glucose metabolism, and translocation of SLC2A4 (GLUT4) to the plasma membrane were dysregulated in both mesenchymal transitions. In the HER2-induced mesenchymal model specifically, dysregulation had enriched metabolism in mitochondria. In the nontumorigenic mesenchymal model, different metabolic pathways were enriched, *e.g.*, regulation of ornithine decarboxylase (ODC), selenocysteine synthesis, selenoamino acid metabolism, and metabolism of polyamines. Considering the differences in nucleoside metabolic pathways, the Golgi system, proteoglycans in cancer, and asparagine N-linked glycosylation, we focused our analysis specifically on metabolic proteins involved in glycan metabolism.

### Metabolic Differences in Two Mesenchymal States Involve Changes to GFPT2 Expression

**Fig. 4:**
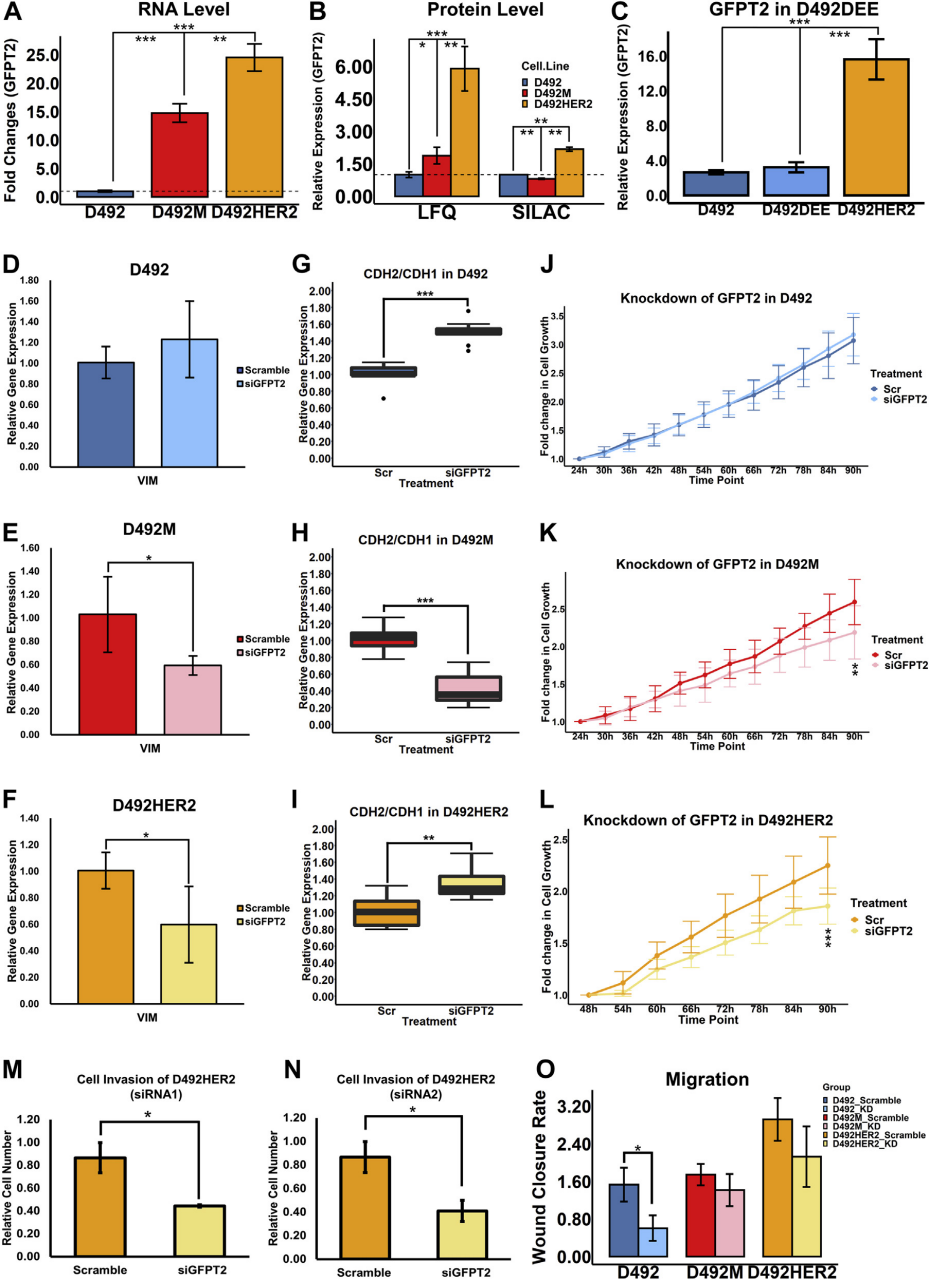
**Expression and functions of GFPT2 in the D492 EMT model.***A*, GFPT2 showed the highest expression in D492HER2 while lowest in D492 on the RNA level; *B,* The protein expression of GFPT2 in the three cell lines suggested highest expression of GFPT2 in D492HER2 confirmed by both LFQ (*left*) and SILAC (*right*); *C*, The GFPT2 level in D492DEE which was the negative control cell line of D492HER2 indicated that the increased expression of GFPT2 was not due to the artifacts from cell handling but the overexpression of HER2. *D*–*F*, siRNA-mediated knockdown of *GFPT2* decreased VIM in both mesenchymal cell lines. *G*–*I*, knockdown of *GFPT2* affected CDH2-to-CDH1 ratios in all cell lines. *J*–*L*, knockdown of *GFPT2* decreased the growth of D492M (*K*) and D492HER2 (*L*) after 90 h from cell seeding. *M* and *N*, D492HER2 cell invasion was decreased after knockdown of *GFPT2* and confirmed by two siRNAs. *O*, D492 cell migration was slowed by the knockdown of *GFPT2*. A decreasing trend was seen in D492M and D492HER2 without significance. ∗*p* < 0.05; ∗∗*p* < 0.01; ∗∗∗*p* < 0.001.

To determine how changes in proteins involved in glycan precursor synthesis come about following EMT, we identified metabolic enzymes with a fold change of two or more in both the LFQ and SILAC datasets across the three cell lines. These targets were grouped into six clusters that spanned several metabolic pathways and included enzymes that have previously been associated with EMT (Fig. 3, *D*–*F* and Table 1). Enzymes closely involved in the metabolism of glycan precursors included PYGB, PGM3, UGDH, PGM2L1, GALNT7, GFPT2, and GALE and were found indiscriminately within different clusters. Altered expression of GALE, UGDH, PGM2L1, and GFPT2 was confirmed using qPCR (Fig. 4*A* and supplemental Fig. S5, *A*–*C*). The biggest difference in RNA expression was detected in GFPT2, consistent with the proteomic analysis (Fig. 4*B*). GFPT2 was not upregulated in D492 transfected with empty vectors (D492DEE) compared with D492HER2 (Fig. 4*C*). GFPT2 is the rate-limiting enzyme in the HBP and catalyzes fructose-6-phosphate to glucosamine-6-phosphate while converting glutamine into glutamate. GFPT2 regulates the availability of precursors for O-GlcNAcylation. Knockdown of *GFPT2* with siRNAs reduced protein O-GlcNAcylation in the D492 cells (supplemental Fig. S5, *D*–*F*).

**Table 1:** Metabolic targets identified by comparing one cell line to another

Protein ID	Protein names	Gene name	KEGG classification	Log2(D492HER2/D492M)	Possible transcription factors	Citation related to EMT
A0A0S2Z4X9	Glutamine-fructose-6-phosphate transaminase 2 isoform 1 (Fragment)	*GFPT2*	Carbohydrate metabolism	1.558	NF-KB; SIRT6; BMP-2	Shaul *et al.*, 2014 (44); Simpson *et al.*, 2012 (50); Szymura *et al.*, 2019 (53); Taparra *et al.*, 2019 (96); Zhang *et al.*, 2018 (55); Zhou *et al.*, 2019 (22)
Q16831	Uridine phosphorylase 1	*UPP1*	Pyrimidine metabolism	1.167	NF-Kb; Oct3/4	Guan *et al.*, 2019 (97); Wehbe *et al.*, 2012 (98)
X5DR03	Glutathione S-transferase mu 1 isoform B (Fragment)	*GSTM1*	Glutathione metabolism	−2.152	Nrf2	n.a
Q53X91	Sulfotransferase (Fragment)	*SULT1E1*	Steroid hormone biosynthesis	−2.383	Nrf2	n.a
Q92626	Peroxidasin homolog	*PXDN*	Oxidoreductases	−3.825	Snail 1; Nrf2	Briem *et al.*, 2019 (16); Sitole and Mavri-Damelin, 2018 (99)

				Log2(D492HER2/D492)		

A0A0S2Z4X9	Glutamine-fructose-6-phosphate transaminase 2 isoform 1 (Fragment)	*GFPT2*	Carbohydrate metabolism	1.827	NF-KB; SIRT6; BMP-2	Shaul *et al.*, 2014 (44); Simpson *et al.*, 2012 (50); Szymura *et al.*, 2019 (53); Taparra *et al.*, 2019 (96); Zhang *et al.*, 2018 (55); Zhou *et al.*, 2019 (22)
Q6FH49	NNMT protein	*NNMT*	Nicotinate and nicotinamide metabolism	1.275	Stat3	Eckert *et al.*, 2019 (100); Shaul *et al.*, 2014 (44)
P16930	Fumarylacetoacetase	*FAH*	Tyrosine metabolism	1.272	CDC5L	n.a
Q6XQN6	Nicotinate phosphoribosyltransferase	*NAPRT*	Nicotinate and nicotinamide metabolism	−1.266	NF-Kb; STAT3; HIF-1a	Lee *et al.*, 2018 (101)
A0A024R6H3	Inositol 1,3,4-triphosphate 5/6 kinase, isoform CRA_a	*ITPK1*	Inositol phosphate metabolism	−1.358	BMP2; TBX2; SNAIL; miR-23b	Bonet *et al.*, 2015 (102)
A0A0B4J2A4	3-ketoacyl-CoA thiolase, mitochondrial	*ACAA2*	Lipid metabolism	−1.737	PPARα; HNF4α	n.a
A0A024RB23	Diacylglycerol kinase	*DGKA*	Lipid metabolism	−1.826	PPARγ; Stat5; AP2, Ets1,SP1	n.a
P11216	Glycogen phosphorylase, brain form	*PYGB*	Starch and sucrose metabolism	−2.018	n.a	Zhang *et al.*, 2018 (55)
P47989	Xanthine dehydrogenase/oxidase	*XDH*	Purine metabolism	−2.908	NF-Y	n.a
Q53FA7	Quinone oxidoreductase PIG3	*TP53I3*	Oxidative stresses and irradiation	−2.911	FOXK2&BAP1	Alonso *et al.*, 2007 (103); Reka *et al.*, 2014 (104)
Q53X91	Sulfotransferase (Fragment)	*SULT1E1*	Steroid hormone biosynthesis	−3.010	Nrf2	n.a
A8YXX4	Glutamine synthetase	*GLUL*	Carbohydrate metabolism	−3.582	ATF4	n.a

				Log2(D492M/D492)		

Q53TK1	Cytochrome P450, family 1, subfamily B, polypeptide 1, isoform CRA_a	*CYP1B1*	Lipid metabolism	3.733	SP1	Kwon *et al.*, 2016 (105); Shaul *et al.*, 2014 (44)
E7EPM6	Long-chain-fatty-acid--CoA ligase 1	*ACSL1*	Lipid metabolism	1.712	SP1	Sánchez-Martínez *et al.*, 2015 (106)
Q6PCE3	Glucose 1,6-bisphosphate synthase	*PGM2L1*	Carbohydrate metabolism	1.422	ZEB1	n.a
O95394	Phosphoacetylglucosamine mutase	*PGM3*	Carbohydrate metabolism	1.393	n.a	n.a
Q6FGJ9	Glutathione S-transferase	*GSTM3*	Glutathione metabolism	1.368	Nrf2	Zhou *et al.*, 2008 (107)
Q86SF2	N-acetylgalactosaminyltransferase 7	*GALNT7*	Glycan biosynthesis and metabolism	1.328	miR-30d/30b; miR-214	n.a
Q6FH49	NNMT protein	*NNMT*	Nicotinate and nicotinamide metabolism	1.181	Stat3	Eckert *et al.*, 2019 (100); Shaul *et al.*, 2014 (44)
Q9BRR6-2	Isoform 2 of ADP-dependent glucokinase	*ADPGK*	Glycolysis/Gluconeogenesis	1.173	n.a	Lee *et al.*, 2016 (108); Song *et al.*, 2018 (109)
O60701	UDP-glucose 6-dehydrogenase	*UGDH*	Carbohydrate metabolism	1.025	SP1	Tang *et al.*, 2016 (110); Vergara *et al.*, 2015 (111)
H0UIA1	Acyl-CoA synthetase short-chain family member 2, isoform CRA_c	*ACSS2*	Carbohydrate metabolism	−1.125	SREBF1/2; HIF; TFEB	Sun *et al.*, 2017 (112)
Q53GQ0	Very-long-chain 3-oxoacyl-CoA reductase	*HSD17B12*	Lipid metabolism	−1.142	n.a	n.a
Q53FA7	Quinone oxidoreductase PIG3	*TP53I3*	Oxidative stresses and irradiation	−1.159	FOXK2&BAP1	Alonso *et al.*, 2007 (103); Reka *et al.*, 2014 (104)
Q14376	UDP-glucose 4-epimerase	*GALE*	Galactose metabolism	−1.458	n.a	n.a
Q6LET6	MGST1 protein (Fragment)	*MGST1*	Glutathione metabolism	−1.522	n.a	Fischer *et al.*, 2015 (113); Shaul *et al.*, 2014 (44)
P11216	Glycogen phosphorylase, brain form	*PYGB*	Starch and sucrose metabolism	−1.611	n.a	Zhang *et al.*, 2018 (55)
P12532	Creatine kinase U-type, mitochondrial	*CKMT1A*	Arginine and proline metabolism	−1.686	LncRNA n335586&miR-924; EVI1&RUNX1	Tanaka and Ogishima, 2015 (114)
A0A0B4J2A4	3-ketoacyl-CoA thiolase, mitochondrial	*ACAA2*	Lipid metabolism	−2.038	PPARα; HNF4α	n.a
B4DLR8	NAD(P)H dehydrogenase [quinone] 1	*NQO1*	Ubiquinone and other terpenoid-quinone biosynthesis	−2.069	Nrf2; NF-Kb	Fischer *et al.*, 2015 (113); Yang *et al.*, 2017 (115)
Q16831	Uridine phosphorylase 1	*UPP1*	Pyrimidine metabolism	−2.303	NF-Kb; Oct3/4	Guan *et al.*, 2019 (97); Wehbe *et al.*, 2012 (98)
A0A024RB23	Diacylglycerol kinase	*DGKA*	Lipid metabolism	−2.730	PPARγ; Stat5; AP2, Ets1,SP1	n.a
A8YXX4	Glutamine synthetase	*GLUL*	Carbohydrate metabolism	−2.741	ATF4	n.a
P47989	Xanthine dehydrogenase/oxidase	*XDH*	Purine metabolism	−3.159	NF-Y	n.a

These targets were with significance (Permutation-based FDR less than 0.05). They were at least twofold changes, comparing D492HER2 to D492M, D492HER2 to D492, and D492M to D492. The fold changes were confirmed by both LFQ and SILAC. The average of Log2 ratios from LFQ and SILAC were reported in this table.

### GFPT2 Influences the EMT Program, Cell Growth, and Cell invasion, and It Is Associated with Claudin-low Breast Cancer

**Fig. 5:**
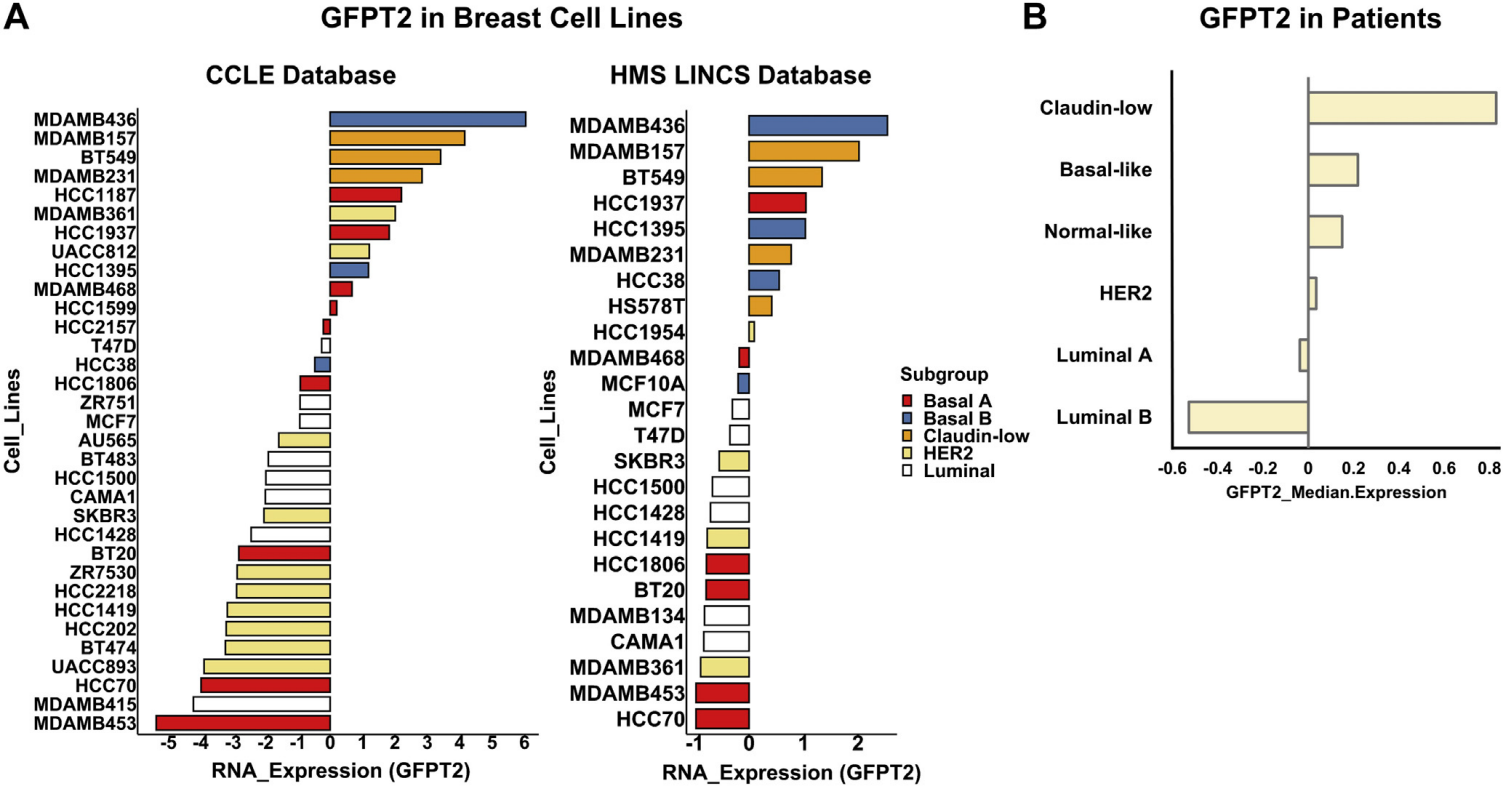
**GFPT2 is higher in basal and claudin-low breast cell lines, and same trend is shown in breast cancer patients.***A*, based on data from an open-source database – CCLE (*left*) (39), GFPT2 was higher in basal and claudin-low cell lines while lowly expressed in HER2-positive and luminal cell lines. The same trend was seen with data from another open-source database – HMS LINCS (*right*) (40). The molecular classification of breast cancer cell lines was based on literature (95). *B*, TCGA data (Breast Cancer (METABRIC, Nature 2012 & Nat Commun 2016)) suggested GFPT2 was expressed highly in claudin-low patients, while its expression was lower in HER2-positive and luminal patients.

The switch between the epithelial marker, E-Cadherin (CDH1), and the mesenchymal maker, N-Cadherin (CDH2), along with the increased expression of vimentin, is hallmark in EMT (46, 47). To interrogate if *GFPT2* knockdown influences the EMT program, we assayed vimentin VIM (Fig. 4, *D*–*F*) and the surface markers CDH1 and CDH2 (Fig. 4, *G*–*I* and supplemental Fig. S6, *A*–*F*). siRNA-mediated knockdown of *GFPT2* decreased the expression of VIM in the mesenchymal cell states and affected the CDH2-to-CDH1 ratios in all three cell lines. Knockdown of *GFPT2* negatively affected cellular growth in both D492M and D492HER2 (Fig. 4, *J*–*L* and supplemental Fig. S7*A*) as well as invasion in D492HER2 (Fig. 4, *M* and *N* and supplemental Fig. S7*B*). Decreasing trends were observed for migration after *GFPT2* knockdown in the three cell lines (Fig. 4*O* and supplemental Fig. S7*C*). No changes were observed in cell morphology (supplemental Fig. S6*G*). To test the generality of these results, we investigated the expression of GFPT2 across different breast cancer subtypes in both cell lines and patients. GFPT2 was not positively associated with HER2-positive but rather claudin-low breast cancer (Fig. 5).

### Hexosamine Biosynthesis Is Upregulated Post Both Mesenchymal Transitions and Dependent Upon GFPT2

**Fig. 6:**
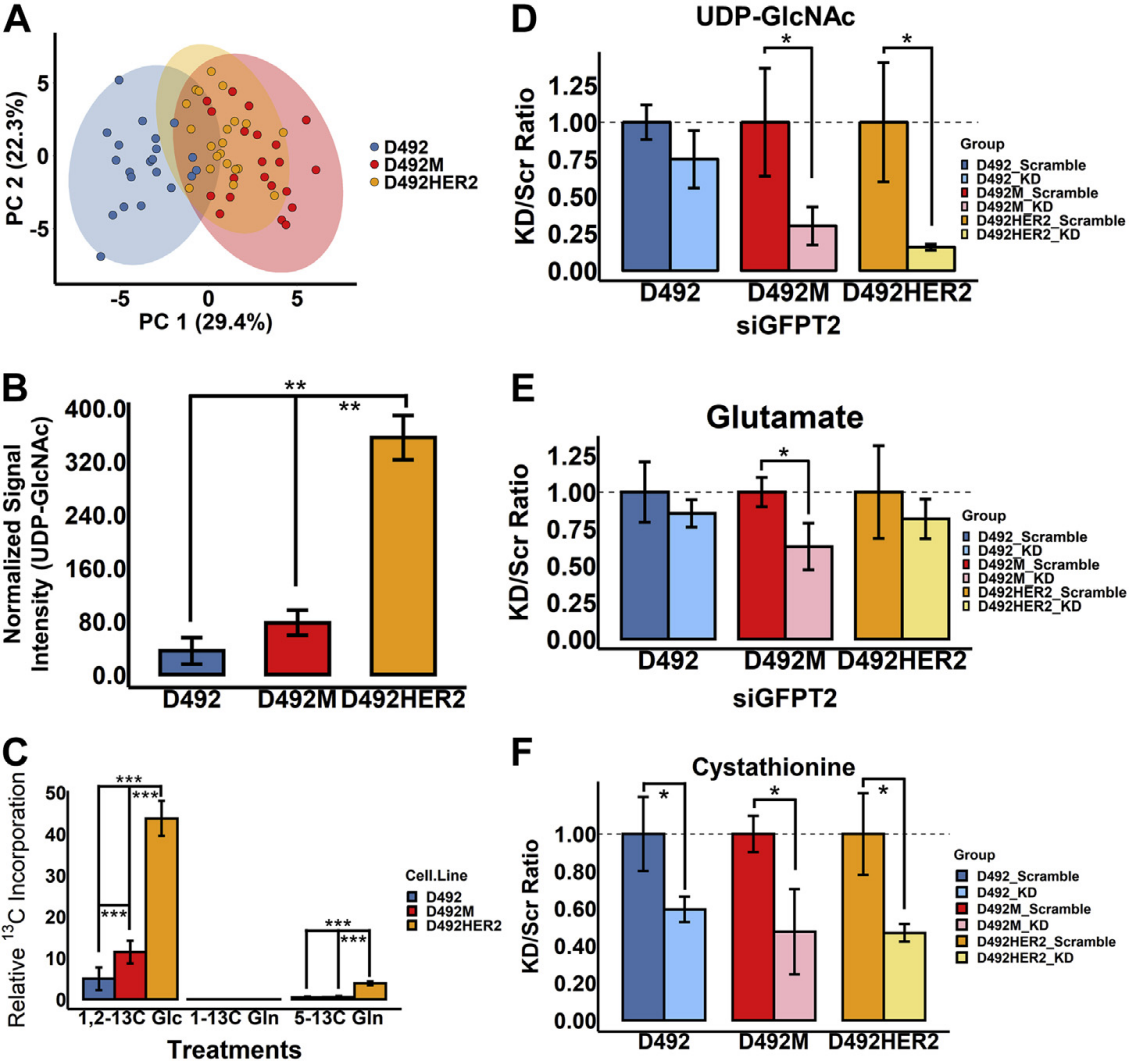
**Metabolomic analyses of the D492 EMT cell model.***A*, the metabolome in D492M and D492HER2 was similar compared with that in D492. *B*, UDP-GlcNAc was expressed higher in D492HER2 compared to the other cell types. *C*, carbon incorporation into UDP-GlcNAc after 6 h’ culture. Carbons from 1,2-^13^C glucose (Glc) were highly incorporated into UDP-GlcNAc, compared to 5-^13^C glutamine (Gln) and 1-^13^C Gln in all three cell lines. No carbon incorporation from 1-^13^C Gln in all three cell lines. Higher rates of carbon incorporation into UDP-GlcNAc from both 1,2-^13^C Glc and 5-^13^C Gln were observed in D492HER2, compared to D492 and D492M. A higher rate of carbon incorporation into UDP-GlcNAc from 1,2-^13^C Glc was observed in D492M compared with D492. *D*, knockdown of *GFPT2* decreased the production of UDP-GlcNAc in both D492M and D492HER2. *E*, a decreasing trend for glutamate with *GFPT2* knockdown was observed in the D492 EMT model. *F*, knockdown of *GFPT2* significantly decreased cystathionine in all three cell types. ∗*p* < 0.05; ∗∗*p* < 0.01; ∗∗∗*p* < 0.001.

We next confirmed changes to glycan metabolic precursors in the D492 model. Metabolomics comparison indicated that D492HER2 was more like D492M than D492 (Fig. 6*A*). UPLC-MS analysis of the glycan precursor metabolites, namely UDP-glucose (UDP-Glc), UDP-glucuronate (UDP-GlcA), N-acetylglucosamine phosphate (GlcNAc-P), and UDP-GlcNAc, showed only significant changes to the GFPT2 product UDP-GlcNAc (Fig. 6*B* and supplemental Fig. S8, *A*–*C*). Relative intracellular concentrations increased by roughly twofold from D492 to D492M and tenfold between D492 and D492HER2. We confirmed altered metabolic activity by monitoring ^13^C isotopologue label enrichment in UDP-GlcNAc from cells grown in media containing 1,2-^13^C glucose, 1-^13^C glutamine, or 5-^13^C glutamine. In context with the relative amount of UDP-GlcNAc, ^13^C enrichment in UDP-GlcNAc from 1,2-^13^C glucose was increased in both D492M and D492HER2. The data indicated an absolute metabolite flux increase into the HBP from glucose that increased *via* D492 < D492M < D492HER2 (Fig. 6*C* and supplemental Fig. S8, *D*–*F*). Little or no enrichment was observed in UDP-GlcNAc from 1-^13^C glutamine. The m + 1 isotopologue in ^13^C enrichment from the 5-^13^C-glutamine was decreased in D492M compared with D492 and D492HER2 (supplemental Fig. S8*F*). Knockdown of *GFPT2* resulted in a clear decrease in the intracellular levels of UDP-GlcNAc in both D492M and D492HER2 (Fig. 6*D*), consistent with reports of its enzymatic function (48).

### GFPT2 Is a Marker for Cellular Oxidative Stress

**Fig. 7:**
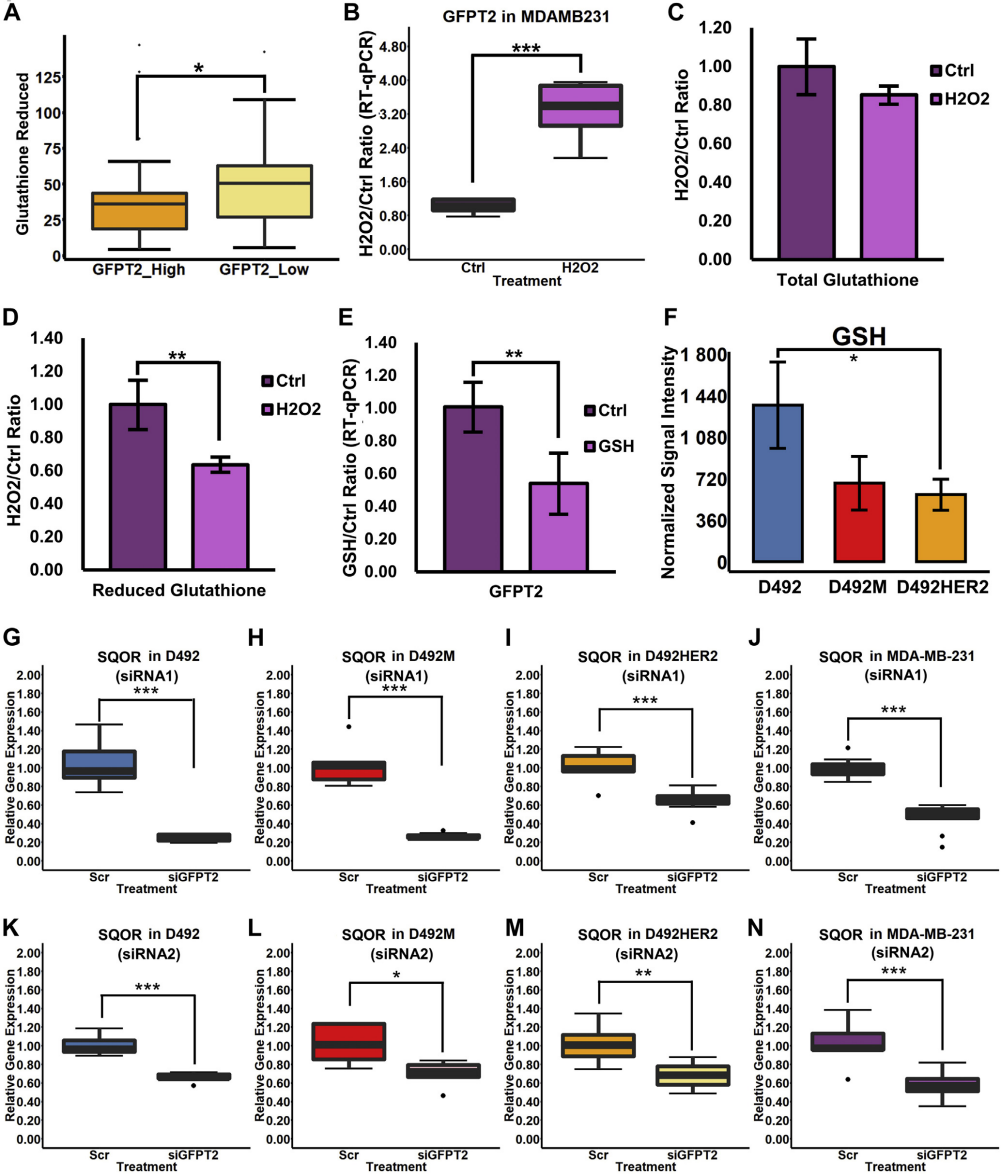
**GFPT2 is a marker for cellular stress.***A*, gene–metabolite correlation analysis of the NCI60 cancer cell line panel indicated a negative correlation between GFPT2 and GSH. *B*, GFPT2 RNA expression was significantly upregulated with 2 μM H_2_O_2_ treatment in MDA-MB-231. *C*, the total glutathione level did not change after H_2_O_2_ treatment. *D*, the GSH level was significantly decreased by the H_2_O_2_ treatment. *E*, treatment with 50 mg/l of GSH significantly downregulated the GFPT2 gene expression in MDA-MB-231. *F*, GSH level was significantly higher in D492 than in D492M and D492HER2. *G*–*N*, SQOR RNA expression was significantly downregulated in D492 (*G*), D492M (*H*), D492HER2 (*I*), and MDA-MB-231 (*J*) by siRNA-mediated knockdown of *GFPT2*, which was confirmed by the second siRNA (*K*–*N*). ∗*p* < 0.05; ∗∗*p* < 0.01; ∗∗∗*p* < 0.001.

The HBP is altered by changes to cellular redox potential (49). GFPT2 may influence GSH through glutamine-derived glutamate (50). In addition to changes to UDP-GlcNAc, knockdown of *GFPT2* resulted in decreased intracellular levels of glutamate (Fig. 6*E*). We similarly noted a decrease in the intracellular cystathionine levels (Fig. 6*F*). Both glutamate and cystathionine can serve as precursors in GSH *de novo* synthesis. A gene–metabolite correlation analysis of the NCI60 cancer cell line panel indicated a negative correlation between GFPT2 and GSH (Fig. 7*A*). No correlation was observed for oxidized glutathione (GSSG), and none was observed for GFPT1 (not shown). Knockdown of *GFPT2*, however, resulted in an increase or no change to glutathione, which we confirmed in the widely studied claudin-low MDA-MB-231 cell line (supplemental Fig. S9, *A*–*H*).

Zitzler *et al.* (51) reported that overexpression of GFPT2 enhances cell survival following H_2_O_2_ treatment. We treated MDA-MB-231 cells with H_2_O_2_ and observed an increase of GFPT2 RNA expression (Fig. 7*B*) with a concomitant reduction to GSH while total glutathione remained unchanged (Fig. 7, *C* and *D*). Furthermore, treatment of the MDA-MB-231 cells with GSH resulted in decreased expression of GFPT2 (Fig. 7*E*), confirming that GFPT2 expression reacts to GSH. The D492 cells possessed higher amounts of GSH than D492M and D492HER2 (Fig. 7*F*), which agreed with the lowest expression of GFPT2 (Fig. 4, *A*–*C*).

Cystathionine is an intermediate metabolite in the transsulfuration pathway and contributes to hydrogen sulfide (H_2_S) and GSH synthesis. Considering that the levels of cystathionine dropped following *GFPT2* knockdown (Fig. 6*F*) while no consistent significant changes to the glutathione level were observed (supplemental Fig. S9, *A*–*H*), we hypothesized that GFPT2 might affect the intracellular H_2_S homeostasis to counteract oxidative stress. SQOR catalyzes the oxidation of H_2_S and glutathione regenerating ubiquinol in the mitochondrial membrane. Following *GFPT2* knockdown, we observed consistent downregulation of SQOR in all four cell lines (Fig. 7, *G*–*N*).

**Fig. 8:**
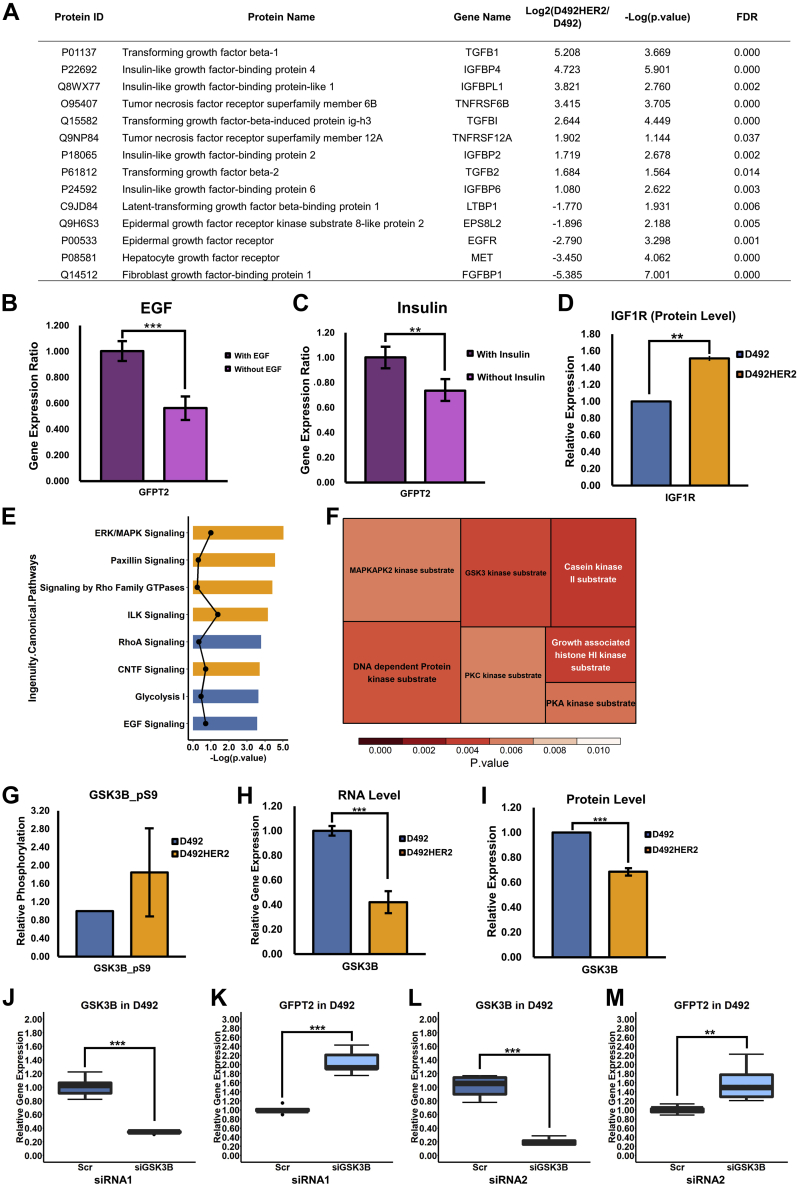
**Signaling regulation of GFPT2.***A*, secretome of D492HER2 and D492 revealed a list of growth factors that secreted differently between these two cell lines (FDR <0.05, Fold change ≥ 2). *B* and *C*, to test the effects of growth factors on GFPT2, we adapted the MDA-MB-231 cells with the FBS-free H14 medium. Removal of EGF and insulin decreased GFPT2 RNA expression in the MDA-MB-231 cell line. *D*, the protein level of IGF1R was higher in D492HER2 than in D492 based on the SILAC proteomic data. *E*, top eight of the Ingenuity Canonical Pathways from the phosphoproteomics data analysis. Pathways activated in D492HER2 were in *orange*, while pathways activated in D492 were in *blue*. *Dots* referred to the absolute value of activation Z-scores. Pathways were listed based on *p* value. *F*, motif enrichment from Perseus (Version 1.6.14.0) suggested a list of kinases behaving differently in D492HER2 compared to D492. *G*, based on the phosphoproteomics analysis, GSK3-β was highly phosphorylated at position serine 9, which inhibits GSK3-β activation in D492HER2 compared with D492. *H* and *I*, RNA (*H*) and protein (*I*) expression of GSK3-β in D492HER2 *versus* D492 indicated the higher abundance of GSK3-β in D492. *J*–*M*, knockdown of *GSK3-β* in D492 increased GFPT2 RNA expression. *J*, knockdown efficiency for *GSK3-β* with the first siRNA. *K*, GFPT2 RNA expression after knockdown of *GSK3-β* in D492 with the first siRNA. *L*, knockdown efficiency for *GSK3-β* with the second siRNA. *M*, GFPT2 RNA expression after knockdown of *GSK3-β* in D492 with the second siRNA. ∗∗*p* < 0.01; ∗∗∗*p* < 0.001.

NF-κB (p65) responds to cell stress (52) and has previously been shown to modulate GFPT2 (53). Knockdown of p65 did, however, not suppress GFPT2 expression in D492HER2 (supplemental Fig. S9, *I* and *J*). Analysis of previously published secretome data from D492 and D492HER2 (54) showed differences in proteins involved in TGF-β, IGF, TNF, and EGF signaling (Fig. 8*A*), with all apart from IGF confirmed to regulate GFPT2 (53, 55, 56). Individual removal of growth factors from MDA-MB-231 growth media resulted in decreased GFPT2 expression following removal of insulin and EGF (Fig. 8, *B* and *C*) consistent with receptor tyrosine kinase (RTK) regulation of GFPT2. Expression of the membrane receptor, IGF1R, was also higher in D492HER2 than in D492 (Fig. 8*D*), supporting the higher activity of IGF signaling in D492HER2. ERK/MAPK are common downstream regulators in the RTK signaling pathways. Phosphoproteomics analysis (supplemental Data 9) confirmed changes in signaling within the ERK/MAPK pathway between D492HER2 and D492 and showed enrichment of the GSK3-β and PKCα substrates (Fig. 8, *E* and *F*). siRNA-mediated knockdown of *GSK3-β* resulted in increased GFPT2 expression (Fig. 8, *G*–*M*).

## Discussion

To define changes to metabolic enzymes associated with EMT phenotypes in the breast gland, we analyzed proteins isolated from three breast cell lines representing three epithelial–mesenchymal states using both LFQ- and SILAC-based proteomics mass spectrometry. We first analyzed the proteomics data to confirm the EMT signature of the D492 EMT model and position the cell lines with respect to other cells derived from breast tissue.

The expression pattern of EMT markers was consistent with previously reported markers of EMT (42, 43). Groger *et al.*, 2012 compared EMT gene expression signatures during cancer progression from 18 independent and published papers and listed the core genes involved in EMT. Good consistency between literature and our datasets was observed in terms of the up/downregulation of these EMT markers and between the two detection methods. LFQ and SILAC were discordant on the expression of IL18 and EPCAM. Results from SILAC were more in agreement with literature reports. The results support the epithelial and mesenchymal phenotypes of the D492 EMT cell model previously reported (14, 15).

The results define the D492 model better in relation to other cell models used to study breast cancer and EMT. Both D492 and D492M clustered within the “basal-like” categories, consistent with the prior classification of D492 (15). D492 clustered with the human breast epithelial cell line MCF10A that, like D492, is derived from a reduction mammoplasty from patients without breast cancer (57). Both are nontumorigenic, and MCF10A, like D492, expresses stem cell-like markers (58, 59, 60). Based on SILAC, D492M was most similar to the tumorigenic cell line MDA-MB-468 (59, 61) originally isolated from a metastatic adenocarcinoma and has been used to study metastasis previously (62). D492HER2 shared more similarities with D492M than D492 based on the proteome clustering but was characterized as claudin-low. Accordingly, D492HER2 thus appears to be an intermediate between D492 and D492M, representing diversion from the natural EMT program upon which tumorigenic properties are gained. Both the LFQ and SILAC data indicated D492HER2 as a “mesenchymal-like/claudin-low” cell type showing the most similarity to the tumorigenic MDA-MB-157 cells originally isolated from metastatic human breast carcinoma (59, 61, 63). Given the basal origin of D492, the relatively small changes to the coverage of the proteome between these cell lines (5–7%), and that the claudin-low phenotype has recently been redefined as a molecular signature found dispersed within the intrinsic breast cancer subtypes (64), these results define D492HER2 as a basal-like cell line with claudin-low phenotypes. The findings position the D492 cell culture model with respect to other commonly researched cell culture models originating from breast tissue based upon their protein content and suggest that the D492 cell model mimics basal-like tumors with D492HER2 prone to claudin-low.

Focusing on metabolism, a variety of metabolic enzymes involved in a diversity of metabolic pathways were significantly altered in EMT according to our data, supporting that EMT is entangled with the metabolic network, *e.g.*, central carbon metabolism including glycolysis and oxidative phosphorylation, pentose phosphate pathway (PPP), and mitochondrial metabolism, lipid metabolism, glutamine metabolism, nucleotide metabolism, and glycan metabolism (65). The upregulation of PGM3, UAP1, and OGT, also components of the HBP in the mesenchymal cells, supports the increased activities of HBP in EMT. GO enrichment analysis and pathway enrichment analysis further indicated differences in glycan metabolism in the D492 EMT model. Multiple transcriptional factors, regulators, and enzymes are influenced by O-GlcNAcylation, and glycans are essential for the formation and function of the extracellular matrix (66, 67). GlcNAcylation plays an essential role in breast cancer metastasis and tumorigenesis (68), in line with the observations that siRNA-mediated knockdown of *GFPT2* imparted negative effects on growth and invasion in the mesenchymal cell lines.

GFPT2 has previously been identified as part of the mesenchymal metabolic signature genes (44) and associated with invasive breast cancer mesenchymal phenotypes on the mRNA level (50). Several studies have focused on the function and regulation of GFPT2 related to its role in modulating O-GlcNAcylation of proteins on account of GFPT2 in producing UDP-GlcNAc (21, 22, 53). GFPT2 has also been shown to counteract oxidative stress (51, 69, 70), although the mechanism behind that remains elusive. Our results demonstrate that GFPT2 affects protein O-GlcNAcylation, regulates the EMT program, and impacts cellular growth and invasion in a cellular subtype-specific manner in breast epithelial cells, which are consistent with the literature mentioned above. Claudin-low breast cancer has recently been redefined and subclassified as a breast cancer subtype (64, 71). GFPT2 was one of the predicted claudin-low signatures in Triple-Negative Breast Cancer reported by Prat *et al.*, 2010 (72). KRAS and LKB1 comutant NSCLC emulates claudin-low breast cancer, and GFPT2 was reported in different studies to be the key player in boosting the malignancy of this type of malignant lung cancer (73, 74). Our results indicate that GFPT2 is a claudin-low breast cancer marker, consistent with the previous finding that D492HER2 with higher expression of GFPT2 belongs to the claudin-low breast cell line. The upregulation of GFPT2 in D492HER2 compared with its negative control cell line D492DEE indicates that the HER2 receptor is somewhat responsible for the GFPT2 overexpression. The lower levels of GFPT2 across HER2-positive cell lines in the public domain however suggest that the HER2 receptor is not the only regulator of GFPT2.

We confirmed increased HBP flux associated with GFPT2 expression. The HBP is central to metabolic rewiring in cancer as it affects glutamine, acetyl-CoA, the nucleotides UTP and UDP, and the glycan substrate UDP-GlcNAc (66). UDP-GlcNAc intracellular concentration increased in accordance with the expression levels of GFPT2 in the D492 cell lines and dropped following *GFPT2* knockdown. Concordantly, ^13^C flux analysis showed increased flux from glucose and glutamine into UDP-GlcNAc. The altered glutamine flux profiles into UDP-GlcNAc are consistent with previously proposed differences in TCA cycle flux in the D492 model on account of altered glutamine utilization following EMT (9). The increased metabolic flux observed alongside enhanced expression of GFPT2 is consistent with a mass action effect and corresponds to GFPT2’s role as a biomarker for glucose uptake independent of GLUT1 (55, 70). ^13^C enrichment from the 5-^13^C-glutamine was negligible but suggestive of flux rerouting in the TCA cycle, particularly in D492M compared with D492 and D492HER2. Specifically, the changes in the m + 1 isotopologue were indicative of alternate carbon contribution to UDP-GlcNAc through citrate-derived cytosolic acetyl-CoA and aspartate and are consistent with more detailed metabolic flux analysis of these cell lines reported in Karvelsson *et al.*, 2021 (23).

In light of increased glutamine uptake following EMT in the D492 cells (9) along with decreased glutamate and cystathionine following *GFPT2* knockdown, we explored if GFPT2 would influence GSH through GFPT2 derived glutamate. Knockdown of *GFPT2* resulted in no change or trends toward increased glutathione that does not support a positive relationship between GFPT2 and glutathione in the four cell lines tested. The regulatory role of GFPT2 on glutathione can however not be excluded merely based on the little impacts of GFPT2 on the net glutathione levels. A negative correlation between GSH and GFPT2 expression was observed across the NCI60 cancer cell line panel. Therefore, even though GFPT2 had limited effects on glutathione, the expression of GFPT2 may be adjusted according to the GSH level. Indeed, H_2_O_2_ treatment increased GFPT2 expression, while GSH treatment had the opposite effect. The D492 cells possessed higher amounts of GSH while GFPT2 expression was low, while the opposite was true for D492M and D492HER2, consistent with increased cell stress in the mesenchymal cells as previously reported (9, 75). The data suggest that redox balance influences GFPT2 expression. High expression of GFPT2 is a marker for oxidative stress important for EMT (76) and breast cancer progression (77).

NF-κB is central to the cellular stress response and is implicated in EMT (52, 78). Following TGF-β/TNFα stimulation, GFPT2 expression is enhanced by the stress regulator NF-κB with which it forms a regulatory feedback loop *via* glycosylation of p65 (21). siRNA-mediated knockdown of p65 did, however, not influence GFPT2 expression in the D492 EMT model. Our results do not exclude that NF-κB induces GFPT2 expression in the stress response with TNFα stimulation. However, it appears that maintenance of GFPT2 expression in claudin-low breast cancer relies on additional factors.

H_2_S originates from the transsulfuration pathway, and knockdown of *GFPT2* resulted in decreases to the pathway intermediate, cystathionine, suggesting that the production of H_2_S could be hampered. SQOR utilizes H_2_S as substrate, and decreased SQOR with *GFPT2* knockdown supports the hampered H_2_S production. However, a solid relationship between GFPT2 and H_2_S could not be established based on limited evidence. Nevertheless, the negative effects of knocking down *GFPT2* on cystathionine and SQOR have connected GFPT2 with H_2_S and further with mitochondrial functions. H_2_S signaling has recently been reported to facilitate EMT (79, 80). However, the exact role of H_2_S in EMT is still controversial (81). Mitochondrial dysfunction is involved both in EMT (82) and in breast cancer (83). A recent study has connected GFPT2 to SLP-2 involved in mitochondrial regulation (70). Understanding the roles of GFPT2 in oxidative stress and H_2_S and mitochondrial homeostasis in mesenchymal cells is beneficial to clinical therapeutic interventions and prognostics. Our results indicate that the effects of GFPT2 on GSH are more complex than can be accounted for by a direct impact on account of limitation to GSH precursors. GFPT2 expression responds to changes in the intracellular redox environment and may alter H_2_S level that impacts SQOR and mitochondria homeostasis. The protective effects of GFPT2 from oxidative stress may thus be attributed in part to changes in H_2_S-SQOR activity, although further research is needed to elucidate this link.

The regulation of GFPT2 is inherently complex and associated with various growth factors and transcriptional regulators, including EGF, TGF-β, TNF, NF-κB, SIRT6, sXBP1, and SLP-2, etc. (53, 55, 56, 70, 84). Mutant KRAS has also been demonstrated to enhance flux into the HBP *via* GFPT2 that is potentiated by loss of LKB1 (74). Our results are consistent with growth factor-mediated regulation of GFPT2 as removal of insulin and EGF from growth media decreased the expression of GFPT2. The effect of the insulin/IGF pathway on GFPT2 has not been reported before. Phosphoproteomics comparison of D492 and D492HER2 also confirmed altered downstream signaling within the ERK/MAPK pathway. Kinase enrichment analysis highlighted ERK/MAPK and components of the Wnt signaling pathway, *i.e.*, GSK3-β and casein kinase. We interrogated GSK3-β on account of its role in responding to oxidative stress (85, 86, 87, 88) and regulation of Wnt signaling of importance to the EMT program (89). GSK3-β phosphorylation was higher in D492HER2 suggesting inactivation of GSK3-β *via* phosphorylation (90). GSK3-β RNA and protein levels were increased in D492, indicating more active GSK3-β kinase activity in D492. Knockdown of *GSK3-β* in the D492 cells upregulated GFPT2 expression suggesting that GSK3-β suppresses GFPT2. GSK3 kinases are regulators of multiple complex biological functions, which can be inhibited by insulin/IGF1 and ERK/MAPK (88, 91, 92, 93). Our data suggest that GFPT2 is under the control of EGF and insulin and downregulated in epithelial D492 by GSK3-β responding to oxidative stress.

**Fig. 9:**
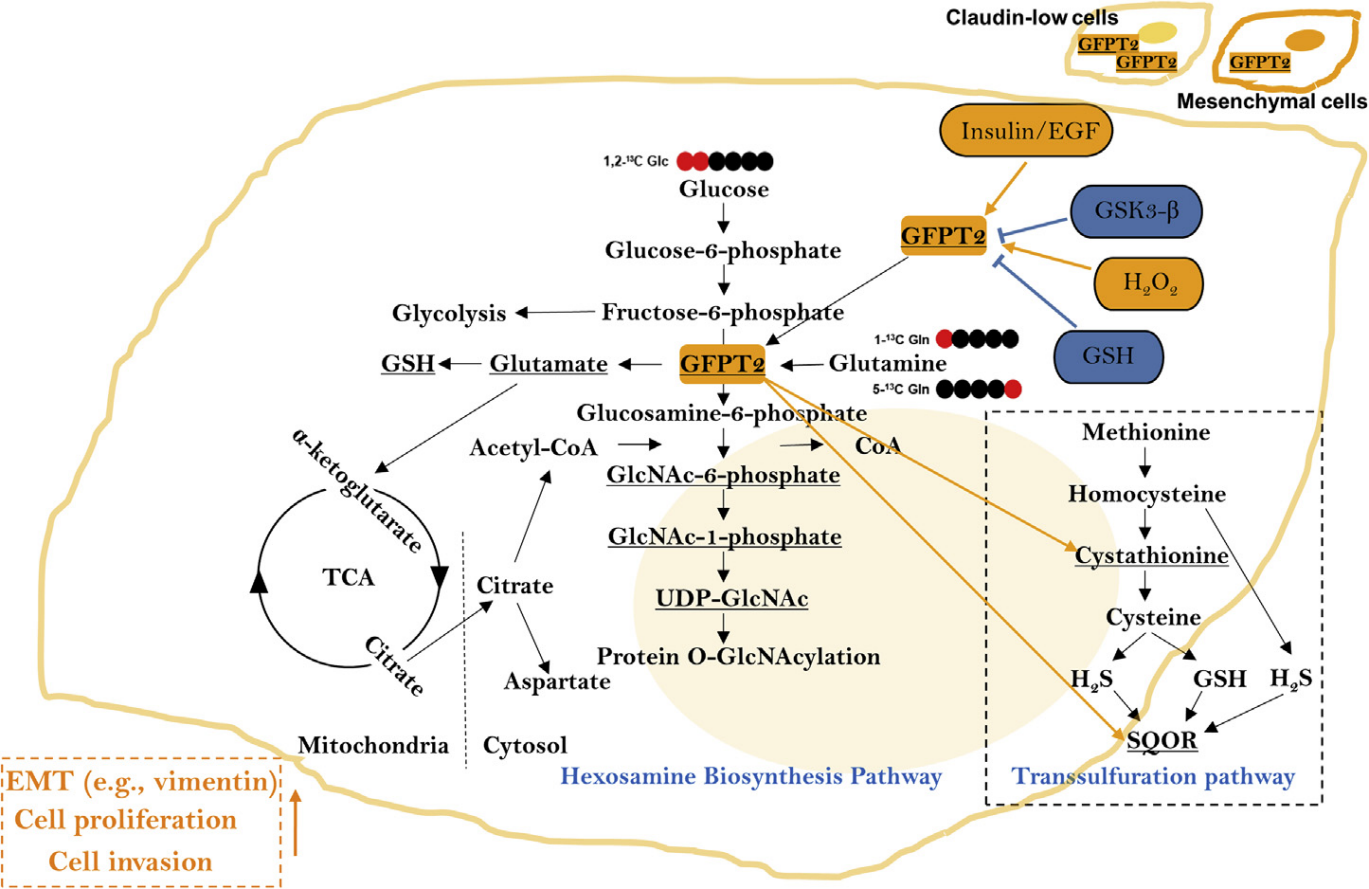
**Summary of the study.** The hexosamine biosynthesis pathway (HBP) and transsulfuration pathway, two main pathways associated with GFPT2 in this study, were illustrated. HBP is closely associated with the central carbon metabolism. The underscored metabolites were measured in this study. We used 1,2-^13^C Glc, 1-^13^C Gln, and 5-^13^C Gln in the ^13^C tracing experiment to trace the carbon flux into UDP-GlcNAc. GFPT2 is the rate-limiting enzyme in the HBP and was upregulated in mesenchymal cells, especially in partial EMT. It affected the HBP flux, EMT program (*e.g.*, VIM), cell growth, and cell invasion, and it was overexpressed in claudin-low breast cancer represented by the D492HER2 cell line. GFPT2 was regulated by oxidative stress (H_2_O_2_ and GSH) and signaling regulators (insulin and EGF, and GSK3-β). Knockdown of *GFPT2* with siRNAs decreased the cystathionine level and SQOR RNA expression in the transsulfuration pathway.

In summary (Fig. 9), GFPT2 is upregulated in breast EMT with higher expression in partial EMT, and knockdown of *GFPT2* in mesenchymal cells decreases the EMT marker VIM and cell proliferation and invasion as well as HBP flux. It is a marker for claudin-low breast cancers and oxidative stress. GFPT2 is under the regulation of the insulin and EGF signaling networks within which GSK3-β imparts a negative effect. Further studies are needed to understand the mechanism of GFPT2 regulating cystathionine, SQOR, the role of GFPT2 in oxidative stress and the transsulfuration pathway, and to confirm the effects of insulin, EGF, and GSK-β on GFPT2 in more cell lines.

## Data Availability

The mass spectrometry proteomics data (LFQ) have been deposited to the ProteomeXchange Consortium *via* the PRIDE (94) partner repository with the dataset identifier PXD025600. The MS/MS spectra have been deposited on MS-Viewer with search keys kkpmzoav00 (LFQ) and ijjdvuqz6w (iBAQ).

The mass spectrometry proteomics data (SILAC) have been deposited to the ProteomeXchange Consortium *via* the PRIDE (94) partner repository with the dataset identifier PXD025858. The MS/MS spectra have been deposited on MS-Viewer with search keys uwpnz5jwk0 (replicate 1), jnuipmemjk (replicate 2), and ztgmyvh5le (replicate 3) and fhlawvfolp (iBAQ).

## Supplemental data

This article contains supplemental data (43).

## Conflict of interest

The authors declare no competing interests.

## Supplemental Data

Supplemental Data 2

Supplemental Data 2_R1

Supplemental Data 2_R2

Supplemental Data 2_R3

Supplemental Data 6

Supplemental Data 6_LFQ

Supplemental Data 6_SILAC

Supplemental Data 9

Supplemental Data 9_Peptides_R1

Supplemental Data 9_Peptides_R2

Supplemental Data 9_Peptides_R3

Supplemental Data 9_PhosphoSites_R1

Supplemental Data 9_PhosphoSites_R2

Supplemental Data 9_PhosphoSites_R3
