# Supplementary material for: Glutamine-Fructose-6-Phosphate Transaminase 2 (GFPT2) Is Upregulated in Breast Epithelial–Mesenchymal Transition and Responds to Oxidative Stress
Source: Mol Cell Proteomics. 2021 Dec 17;21(2):100185. doi: 10.1016/j.mcpro.2021.100185 (PMC8803663; doi:10.1016/j.mcpro.2021.100185)
Supplement: Supplemental Figures S1–S9 and Tables S1, S2 [file mmc21.pdf]

Supplemental Data

Glutamine-fructose-6-phosphate transaminase 2 (GFPT2) is upregulated in breast epithelial-mesenchymal transition and responds to oxidative stress

Qiong Wang[1], Sigurdur Trausti Karvelsson[1], Aristotelis Kotronoulas[1], Thorarinn Gudjonsson[2], Skarphedinn Halldorsson[1,3], Ottar Rolfsson[1]

[1]Center for Systems Biology, Biomedical Center, Faculty of Medicine, School of Health Sciences, University of Iceland, Sturlugata 8, 101 Reykjavik, Iceland.

[2]Stem Cell Research Unit, Biomedical Center, Department of Anatomy, Faculty of Medicine, School of Health Sciences, University of Iceland, Vatnsmyrarvegi 16, 101 Reykjavík, Iceland.

[3]Institute for Surgical Research, Vilhelm Magnus Laboratory, Oslo University Hospital, Oslo, Norway.

Corresponding author information

Ottar Rolfsson[1]; ottarr@hi.is; Center for Systems Biology, Biomedical Center, Faculty of Medicine, School of Health Sciences, University of Iceland, Sturlugata 8, 101 Reykjavik, Iceland.

List of Materials

Supplemental Figures

Figure S1: LFQ *versus* SILAC and the proteome of the D492 EMT cell model.

Figure S2: Evaluate the D492 EMT model based on EMT markers from database dbEMT2.

Figure S3: GO annotation of biological processes and KEGG pathway enrichment of differently expressed proteome between two cell lines.

Figure S4: Interaction network of the changed proteome in metabolism.

Figure S5: Glycan metabolic enzymes and the siRNA-mediated knockdown efficiency and O-GlcNAcylation function of GFPT2.

Figure S6: CDH1/CDH2 expression and phenotypes of D492, D492M, and D492HER2 after siRNA-mediated knockdown of *GFPT2*.

Figure S7: Cell images of D492, D492M, and D492HER2 after knockdown of *GFPT2* in proliferation, migration, and invasion.

Figure S8: Metabolomic levels of the glycan precursors and ^{13}C tracing from glucose and glutamine.

Figure S9: Glutathione levels with *GFPT2* knockdown and GFPT2 RNA expression after siRNA-mediated knockdown of *NF-κB* (p65).

Supplemental Tables

Table S1: Primers in this study.

Table S2: Summary of the LFQ and SILAC proteomic datasets.

Supplemental Data

Supplemental Data 1: MaxQuant output of the LFQ proteomic experiment including protein quantification and peptide information.

Supplemental Data 2: MaxQuant output of the SILAC proteomic experiment including protein quantification and peptide information.

Supplemental Data 3: Signature proteins for D492, D492M, and D492HER2.

Supplemental Data 4: Comparison of the EMT markers between literature and the current study.

Supplemental Data 5: Comparison of the EMT markers between the public database dbEMT2 and the current study.

Supplemental Data 6: MaxQuant output of the iBAQ quantification from the LFQ and SILAC proteomic experiments including protein quantification and peptide information.

Supplemental Data 7: Differently expressed proteins comparing D492, D492M, and D492HER2.

Supplemental Data 8: The enriched KEGG pathways and proteins involved in each pathway comparing D492, D492M, and D492HER2.

Supplemental Data 9: MaxQuant output of the SILAC phosphoproteomic experiment including protein quantification, peptides, and phosphosite information.

Supplemental Figures

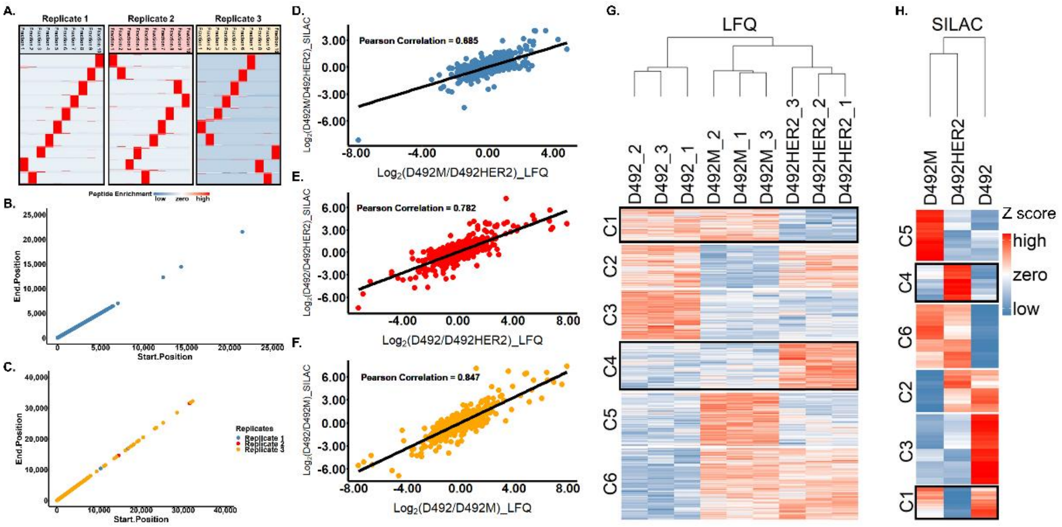

***Figure S1*. LFQ *versus* SILAC and the proteome of the D492 EMT cell model**. **(A)** The peptides were well fractionated into 10 fractions for the three replicates in the SILAC proteomic experiment. **(B)** SILAC, which covered 35,000 amino acids length, had a bigger coverage than LFQ in which only 25,000 amino acids were covered for the peptide detection **(C)**. **(D-F)** The consistency between LFQ and SILAC proteomics datasets regarding ratios of D492M and D492HER2 **(D)**, D492 and D492HER2 **(E)**, and D492 and D492M **(F)**. Pearson comparison coefficients were labeled. Identified and quantified proteins in both the LFQ and SILAC datasets were used for comparison. Proteins needed to be detected in at least two out of three replicates in the LFQ dataset while detected with at least two ratios in three SILAC replicates. Log2 ratios were plotted in the scatter plot with LFQ on the x-axis and SILAC on the y-axis. **(G-H)** “Signature proteome” among D492, D492M, and D492HER2. Proteomic data based on both LFQ **(G)** and SILAC **(H)** have clustered D492M and D492HER2 together. ‘Signature proteins’ for D492 (C3 and C6), D492M (C2 and C5), and D492HER2 (C1 and C4) were defined as protein groups differentiating different cell types. Protein clusters (C1 and C4) were outlined as “Signature Proteins” in D492HER2. The heatmap of the LFQ proteome was plotted using proteins with significant differences (LFQ, ANOVA, and Permutation-based FDR < 0.05), while the heatmap of the SILAC proteome was plotted using the SILAC data (Coefficient of Variation of SILAC ratios < 0.1) **(Supplemental data 3)**. LFQ: Label-free quantification; SILAC: Stable isotope labeling by amino acids in cell culture.

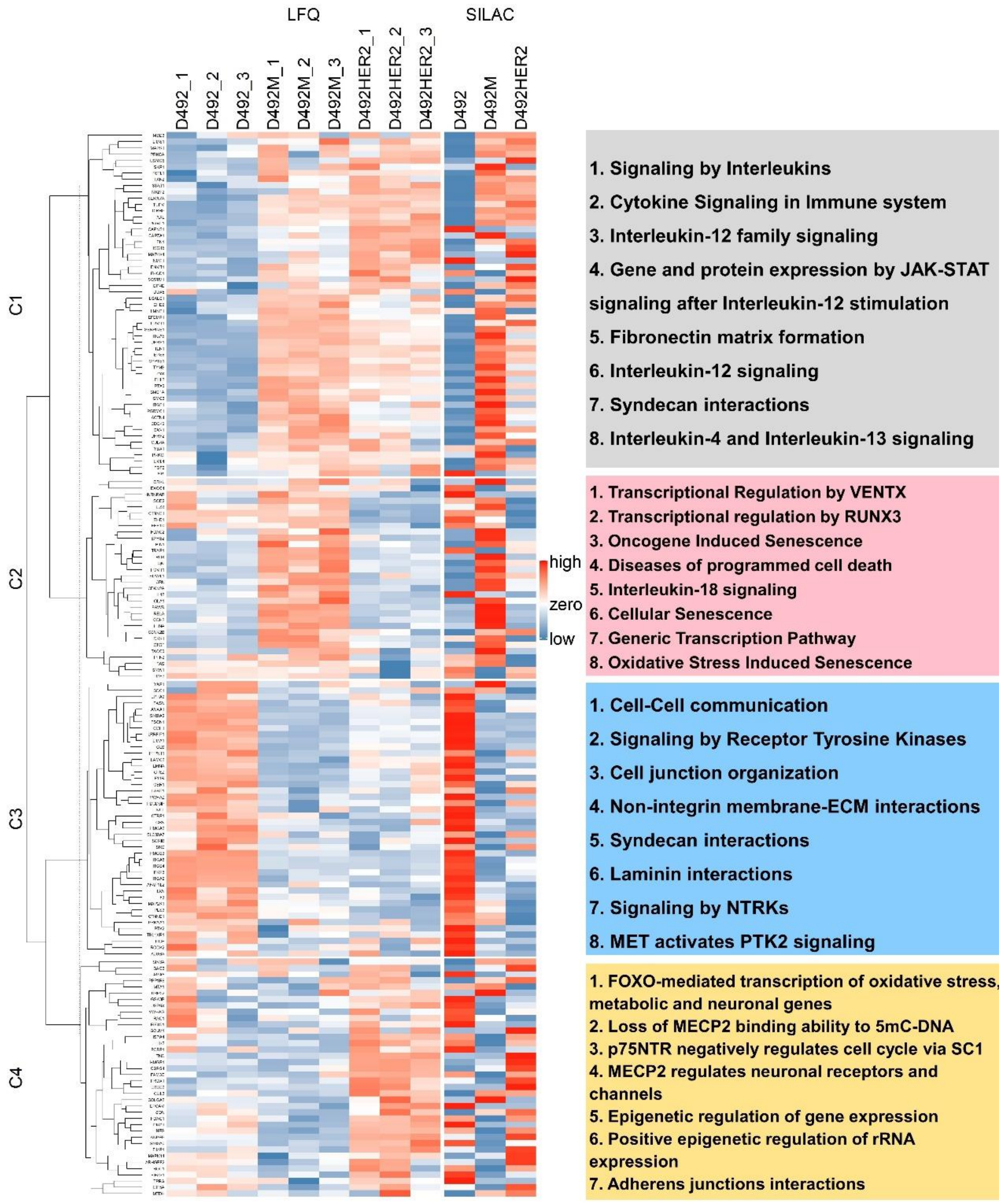

***Figure S2*. Evaluate the D492 EMT model based on EMT markers from database dbEMT2.** Since the EMT program cannot be defined with limited markers, to comprehensively present the expression levels of EMT markers in different cell lines, we plotted all EMT markers detected in this study based on an open EMT database: dbEMT2 (43). EMT markers detected in the study were clustered into four groups. Pathways were enriched for each cluster based on the Reactome pathway database (Version 72), and top pathways in each group (FDR < 0.01) were listed on the right side. The proteins in each cluster were listed in **Supplemental data 5** along with the relative information.

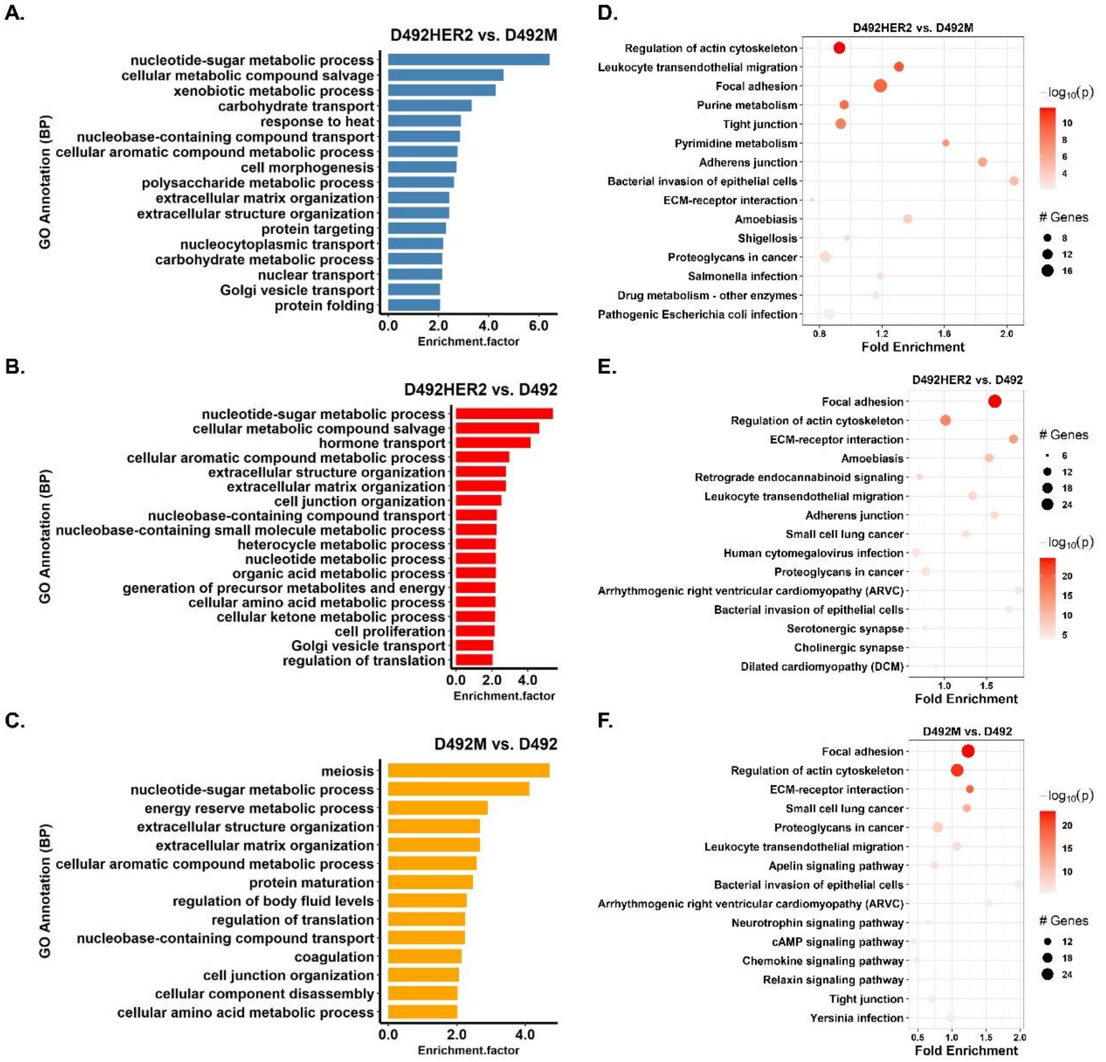

Figure S3. **GO annotation of biological processes and KEGG pathway enrichment of differently expressed proteome between two cell lines.** Annotation of the differences in biological processes (BP) between D492HER2 and D492M **(A)**, D492HER2 and D492 **(B)**, and D492M and D492 **(C)**. The whole proteome from the SILAC dataset was used as background. The enriched GO annotation terms with enrichment factor more than 2 for BPs were plotted in descending order (Fisher exact test, Benjamin-Hochberg FDR < 0.02). **Supplemental data 7** was used for the GO annotation: Student T-test, Permutation-based FDR < 0.05 for LFQ; One sample T-test, p value of SILAC ratios < 0.05. **(D)** KEGG pathways which were involved in cell structure, migration, adhesion, nucleotide metabolism, invasion, proteoglycans in cancer, and more. were differently enriched in D492HER2 *versus* D492M. **(E)** Similar to D492HER2 *versus* D492M, KEGG pathways differently enriched in D492HER2 *versus* D492 were cell structure, migration, adhesion, invasion, and proteoglycans in cancer. **(F)** KEGG pathways differently enriched in D492M *versus* D492 were not only involved in adhesion and migration but also in several signaling pathways. Proteins involved in each KEGG pathway were reported in **Supplemental data 8**.

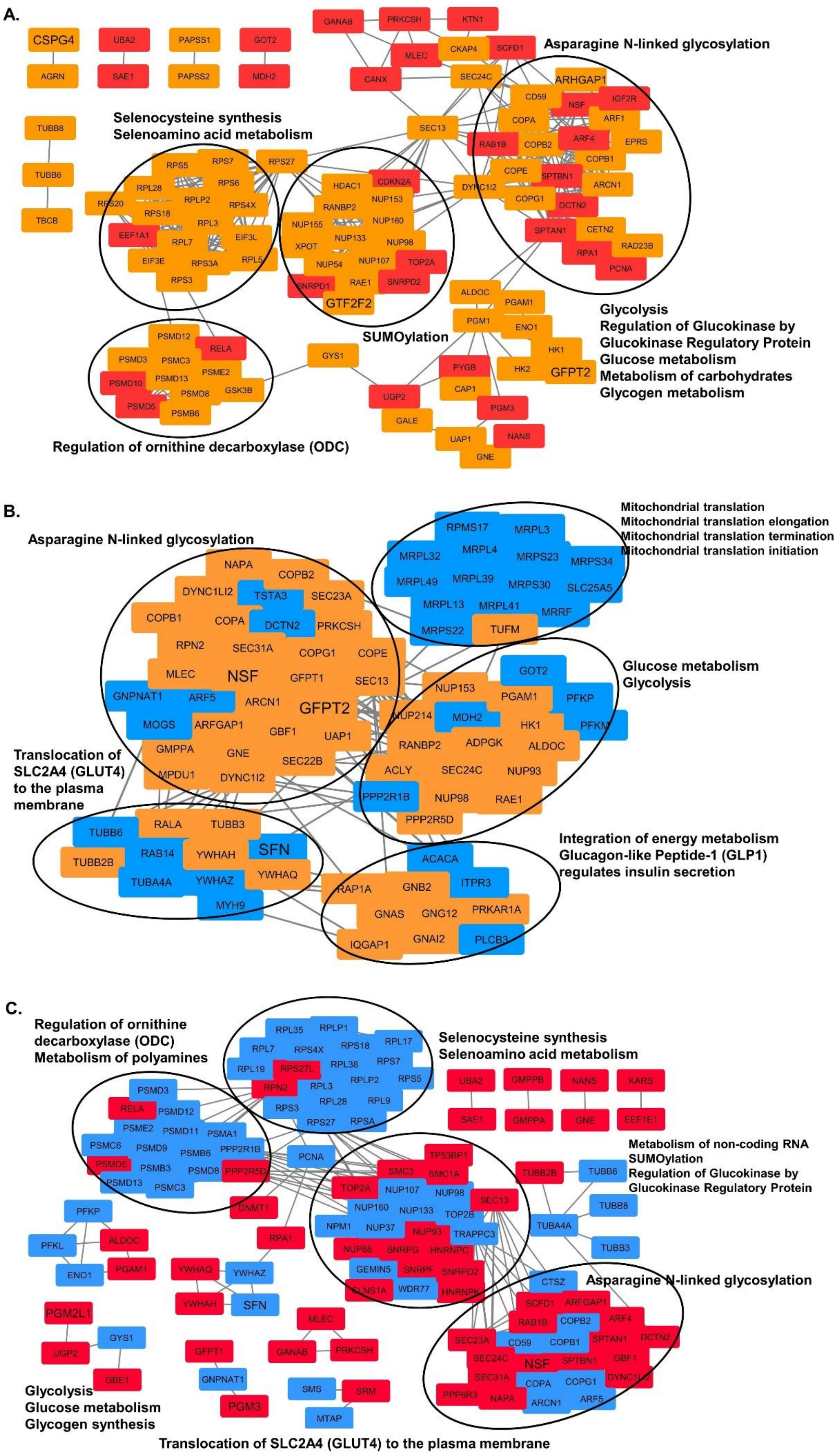
A.
CSPG4
AGRN
UBA2
SAE1
PAPSS1
PAPSS2
GOT2
MDH2
TUBB8
TUBB6
TBCB
GANAB
PRKCSH
KTN1
MLEC
CKAP4
SCFD1
CANX
SEC24C
Asparagine N-linked glycosylation
ARHGAP1
CD59
IGF2R
NSF
ARF1
COPA
SEC13
RAB1B
COPB2
ARF4
EPRS
COPB1
COPE
SPTBN1
ARCN1
DYNC1I2
COPG1
DCTN2
SPTAN1
CETN2
RAD23B
RPA1
PCNA
Selenocysteine synthesis
Selenoamino acid metabolism
RPS5
RPS7
RPS27
RPL28
RPS6
RPLP2
RPS4X
RPS20
RPS18
RPL3
EEF1A1
RPL7
EIF3L
EIF3E
RPS3A
RPL5
RPS3
HDAC1
CDKN2A
RANBP2
NUP153
NUP155
NUP160
XPOT
NUP133
NUP98
NUP54
NUP107
TOP2A
SNRPD1
RAE1
SNRPD2
GTF2F2
SUMOylation
ALDOC
PGAM1
PGM1
ENO1
HK1
HK2
GFPT2
PYGB
CAP1
UGP2
GALE
PGM3
UAP1
NANS
GNE
GYS1
Glycolysis
Regulation of Glucokinase by
Glucokinase Regulatory Protein
Glucose metabolism
Metabolism of carbohydrates
Glycogen metabolism
PSMD12
RELA
PSMD3
PSMC3
PSME2
PSMD10
PSMD13
GSK3B
PSMD8
PSMD5
PSMB6
Regulation of ornithine decarboxylase (ODC)
B.
Asparagine N-linked glycosylation
NAPA
COPB2
DYNC1LI2
TSTA3
SEC23A
COPB1
COPA
DCTN2
PRKCSH
RPN2
SEC31A
COPG1
COPE
MLEC
NSF
GFPT1
SEC13
GNPNAT1
ARF5
ARCN1
GFPT2
MOGS
ARFGAP1
GBF1
UAP1
GMPPA
GNE
SEC22B
MPDU1
DYNC1I2
Mitochondrial translation
Mitochondrial translation elongation
Mitochondrial translation termination
Mitochondrial translation initiation
RPMS17
MRPL3
MRPL4
MRPL32
MRPS23
MRPS34
MRPL49
MRPL39
MRPS30
SLC25A5
MRPL13
MRPL41
MRRF
MRPS22
TUFM
Glucose metabolism
Glycolysis
GOT2
NUP153
PGAM1
PFKP
NUP214
MDH2
HK1
PFKM
RANBP2
ADPGK
ALDOC
ACLY
SEC24C
NUP93
PPP2R1B
NUP98
RAE1
PPP2R5D
Translocation of
SLC2A4 (GLUT4)
to the plasma
membrane
TUBB6
RALA
TUBB3
RAB14
YWHAH
SFN
TUBB2B
TUBA4A
YWHAZ
YWHAQ
MYH9
Integration of energy metabolism
Glucagon-like Peptide-1 (GLP1)
regulates insulin secretion
ACACA
RAP1A
GNB2
ITPR3
GNAS
GNG12
PRKAR1A
IQGAP1
GNAI2
PLCB3
C.
Regulation of ornithine
decarboxylase (ODC)
Metabolism of polyamines
RPL35
RPLP1
RPL17
RPL7
RPS4X
RPS18
RPS7
RPL19
RPS27L
RPL38
RPN2
RPL3
RPLP2
RPS5
RPS3
RPL28
RPL9
RPS27
RPSA
Selenocysteine synthesis
Selenoamino acid metabolism
UBA2
GMPPB
NANS
KARS
SAE1
GMPPA
GNE
EEF1E1
PSMD3
RELA
PSMD12
PSME2
PSMD11
PSMA1
PSMC6
PSMD9
PSMB6
PPP2R1B
PSMD5
PSMB3
PSMD8
PPP2R5D
PSMD13
PSMC3
PCNA
DNMT1
Metabolism of non-coding RNA
SUMOylation
Regulation of Glucokinase by
Glucokinase Regulatory Protein
TP53BP1
SMC3
SMC1A
TOP2A
NUP107
NUP98
SEC13
NUP160
NUP133
TOP2B
NPM1
NUP37
NUP93
TRAPPC3
NUP88
SNRPG
HNRNPC
GEMIN5
SNRPF
SNRPD2
CLNS1A
WDR77
HNRNPK
TUBB2B
TUBB6
TUBA4A
TUBB8
TUBB3
RPA1
PFKP
PFKL
ALDOC
ENO1
PGAM1
YWHAQ
YWHAZ
YWHAH
SFN
PGM2L1
GYS1
UGP2
GBE1
GFPT1
GNPNAT1
PGM3
MLEC
GANAB
PRKCSH
SMS
SRM
MTAP
CTSZ
Asparagine N-linked glycosylation
SCFD1
ARFGAP1
RAB1B
COPB2
ARF4
SEC23A
CD59
COPB1
SPTAN1
DCTN2
SEC24C
NSF
SPTBN1
GBF1
SEC31A
COPA
COPG1
DYNC1I2
PPP6R3
NAPA
ARCN1
ARF5
Glycolysis
Glucose metabolism
Glycogen synthesis
Translocation of SLC2A4 (GLUT4) to the plasma membrane

Figure S4. **Interaction network of the changed proteome in metabolism. (A)** The Reactome metabolic pathways differently enriched in D492HER2 *versus* D492M along with the proteins involved were plotted. Proteins marked in orange were upregulated in D492HER2, and proteins marked in red were upregulated in D492M. **(B)** The Reactome metabolic pathways differently enriched in D492HER2 *versus* D492 along with the proteins involved were plotted. Proteins marked in orange were upregulated in D492HER2, and proteins marked in blue were upregulated in D492. **(C)** The Reactome metabolic pathways differently enriched in D492M *versus* D492 along with the proteins involved were plotted. Proteins marked in red were upregulated in D492M, and proteins marked in blue were upregulated in D492. The gene names with enlarged labels were proteins with at least 2-fold differences between two cell lines. The proteins involved in enriched metabolic pathways (FDR < 0.05) were connected and clustered in STRING (Version 11.0; k-means clustering, minimum required interaction scores: medium confidence 0.400) and visualized in Cytoscape (version 3.5.1/Version 3.6.1). The pathways were enriched with all differentially expressed proteins **(Supplemental data 7)** between cell lines by Reactome Pathway Enrichment Analysis (Version 65, 67, and 72).

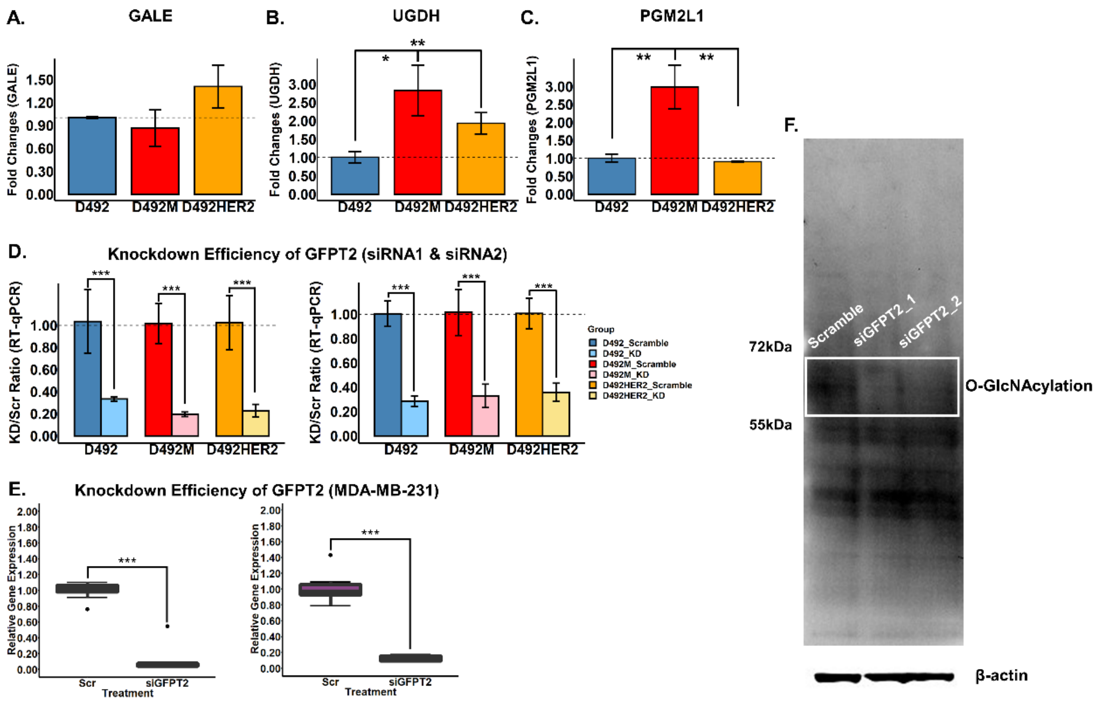

Figure S5. **Glycan metabolic enzymes and the siRNA-mediated knockdown efficiency and O-GlcNAcylation function of GFPT2. (A)** The RNA level of GALE was higher in D492HER2 than in D492, which was contradictory to the protein level **(Fig. 3D)**. **(B-C)** The RNA expressions of UGDH and PGM2L1 were consistent with the protein levels in the three D492 cell lines **(Fig. 3D)**. **(D)** The knockdown efficiency of *GFPT2* with two different siRNAs in the three D492 cell lines showed that both siRNAs had relatively 80 % knockdown efficiency. **(E)** The knockdown efficiency was similar if not better in MDA-MB-231 compared to the D492 cell lines, with more than 80 %. **(F)** The protein O-GlcNAcylation was decreased with *GFPT2* knockdown. It was confirmed by the western blot of protein O-GlcNAcylation in the D492 cells treated with two *GFPT2*-targeting siRNAs, using β-actin as the loading control. *****: $p < 0.05$; ******: $p < 0.01$; *******: $p < 0.001$. GALE: UDP-glucose 4-epimerase; UGDH: UDP-glucose 6-dehydrogenase; PGM2L1: Glucose 1,6-bisphosphate synthase; GFPT2: Glutamine-fructose-6-phosphate transaminase 2.

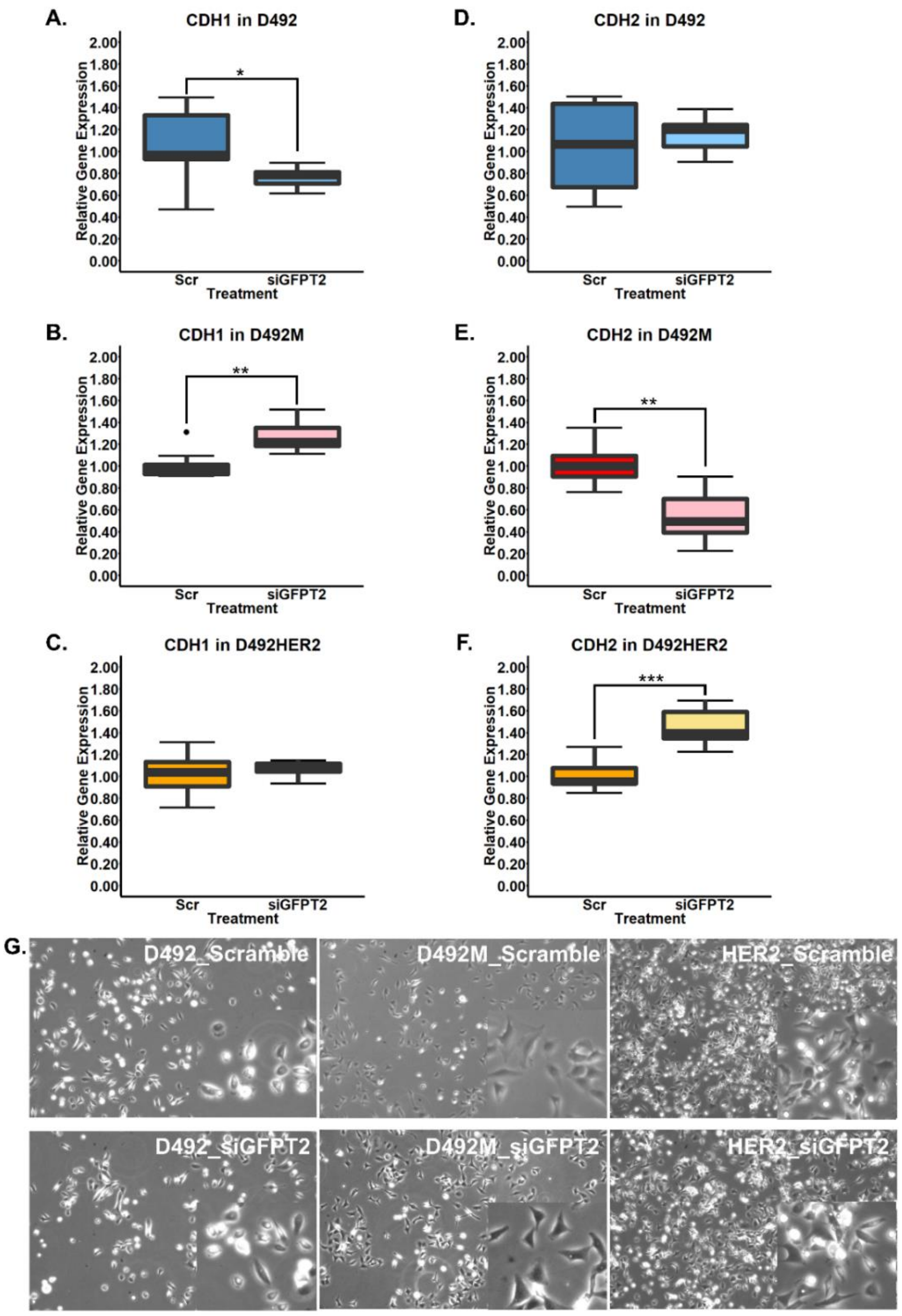

Figure S6. **CDH1/CDH2 expression and phenotypes of D492, D492M, and D492HER2 after siRNA-mediated knockdown of *GFPT2*. (A-C)** Knockdown of *GFPT2* decreased CDH1 in D492 **(A)** while increased CDH1 in D492M **(B)**. **(D-F)** Knockdown of *GFPT2* decreased CDH2 in D492M **(E)** while increased CDH2 in D492HER2 **(F)**. CDH1 was highly expressed in D492 **(Fig. 1C)**, and a decrease in its expression was observed following *GFPT2* knockdown. Similarly, CDH2 was highly expressed in D492M **(Fig. 1C)** but decreased upon knockdown of *GFPT2*. Both CDH1 and CDH2 were lower in D492HER2 compared to D492 and D492M, respectively **(Fig. 1C)**. *GFPT2* knockdown increased the CDH1 expression in D492M and the CDH2 expression in D492HER2. **(G)** Cell phenotype was not noticeably changed in all D492 cell lines with *GFPT2* knockdown. *****: $p < 0.05$; ******: $p < 0.01$; *******: $p < 0.001$. CDH1: Cadherin-1; CDH2: Cadherin-2; GFPT2: Glutamine-fructose-6-phosphate transaminase 2.

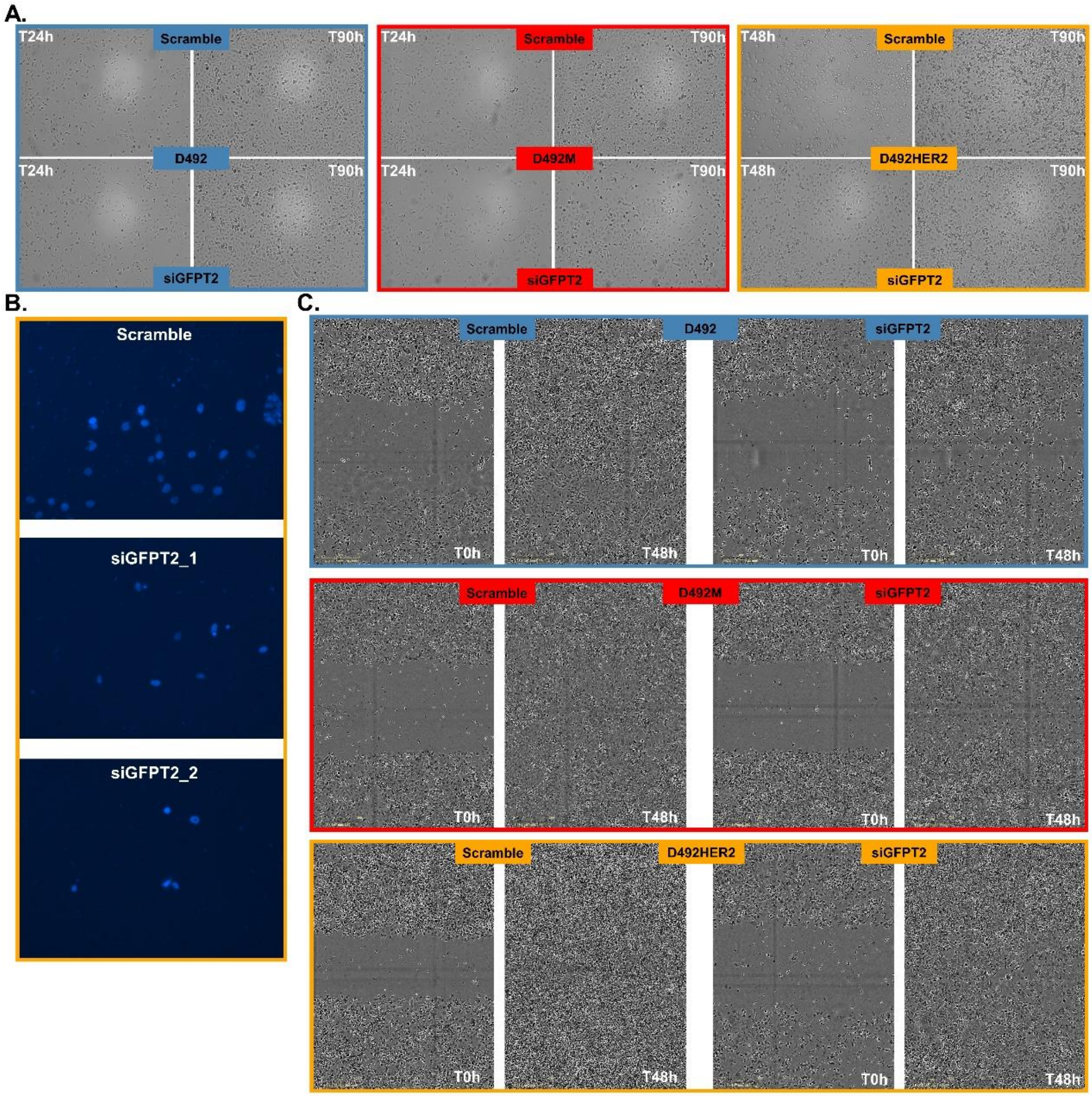

Figure S7. **Cell images of D492, D492M, and D492HER2 after knockdown of *GFPT2* in proliferation, migration, and invasion. (A)** Cell proliferation assay. The representative photos of D492, D492M, and D492HER2 cells with scramble negative control and *GFPT2*-targeting siRNA-mediated treatments in bright-field 24 (48) hours and 90 hours after cell seeding. **(B)** Invasion Transwell assay. The representative photos of D492HER2 with scramble and two *GFPT2*-targeting siRNA-mediated treatments. The invaded cells that passed through the filters were stained with DAPI. **(C)** Wound healing assay. The representative photos of D492, D492M, and D492HER2 cells with scramble and *GFPT2*-targeting siRNA-mediated treatments in phase-contrast at T0 and 48 hours after wound scratching. DAPI: 4’,6-diamidino-2-phenylindole; GFPT2: Glutamine-fructose-6-phosphate transaminase 2.

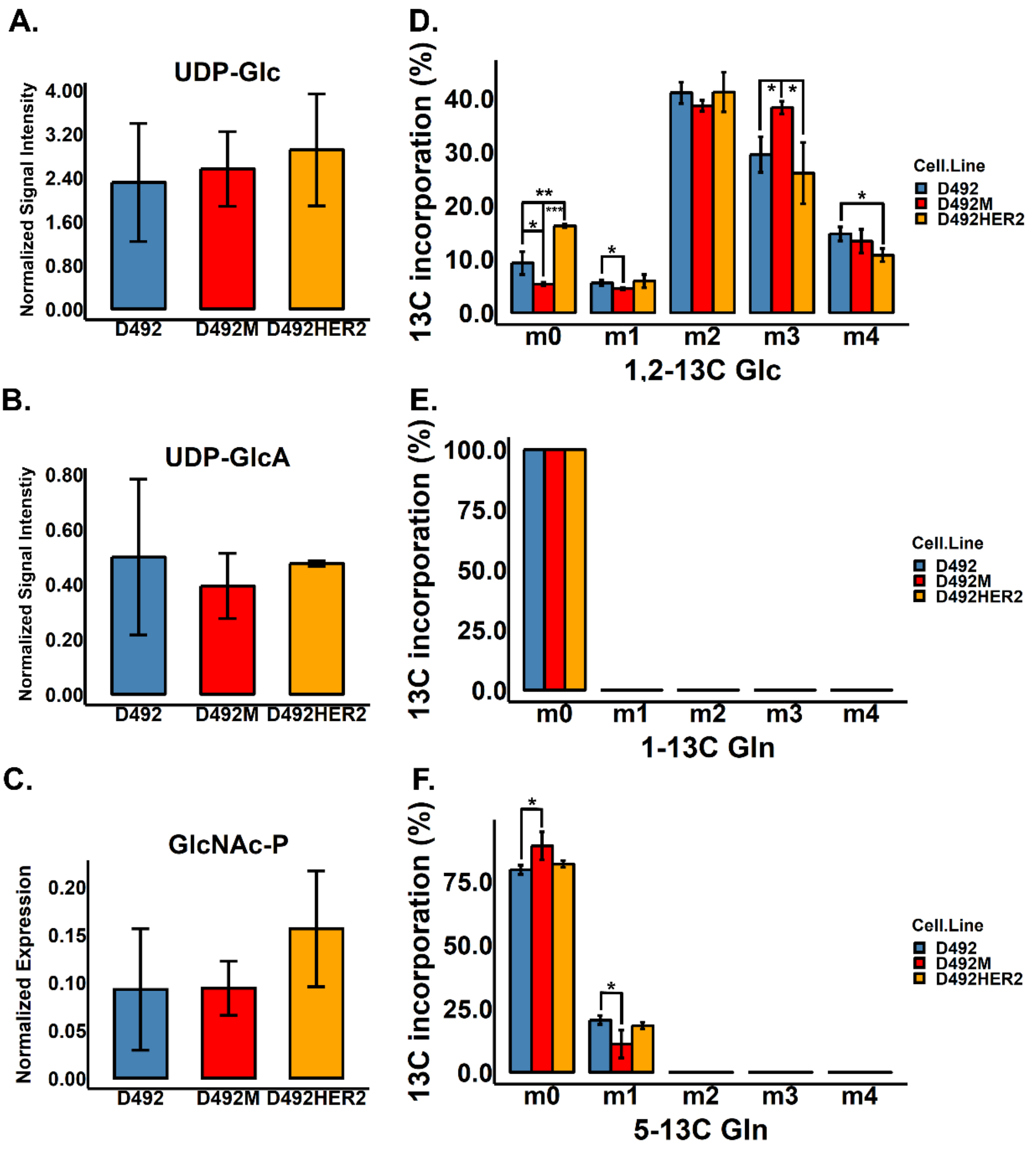

Figure S8. **Metabolomic levels of the glycan precursors and ^{13}C tracing from glucose and glutamine. (A-C)** No significant differences in UDP-Glc **(A)**, UDP-GlcA **(B)**, and GlcNAc-P **(C)** were observed among the 492 cell lines. **(D-F)** The percentage of ^{13}C incorporation after 6 hours' cell culture with 1,2-^{13}C Glc **(D)**, 1-^{13}C Gln **(E)**, and 5-^{13}C Gln **(F)** in all cell lines. **(D)** UDP-GlcNAc molecules were most with two ^{13}C labels, followed by three ^{13}C labels in all cell types when culturing with 1,2-^{13}C Glc. The percentage of UDP-GlcNAc with no ^{13}C incorporation from 1,2-^{13}C Glc was higher in D492HER2 than in D492. D492M had the least percentage of UDP-GlcNAc without labels. The percentage of UDP-GlcNAc with three labels from 1,2-^{13}C Glc was the highest in D492M. **(E)** No ^{13}C incorporation from 1-^{13}C Gln was observed in all cell lines. **(F)** UDP-GlcNAc was most with no ^{13}C label in all cell lines when cultured with 5-^{13}C Gln. The percentage of UDP-GlcNAc with no label from 5-^{13}C Gln was higher in D492M than in D492, while D492M had a significantly lower percentage of UDP-GlcNAc with one label from 5-^{13}C Gln as compared to D492. *****: $p < 0.05$; ******: $p < 0.01$; *******: $p < 0.001$. UDP-Glc: UDP-glucose; UDP-GlcA: UDP-glucuronate; GlcNAc-P: N-acetylglucosamine phosphate; Gln: Glutamine.

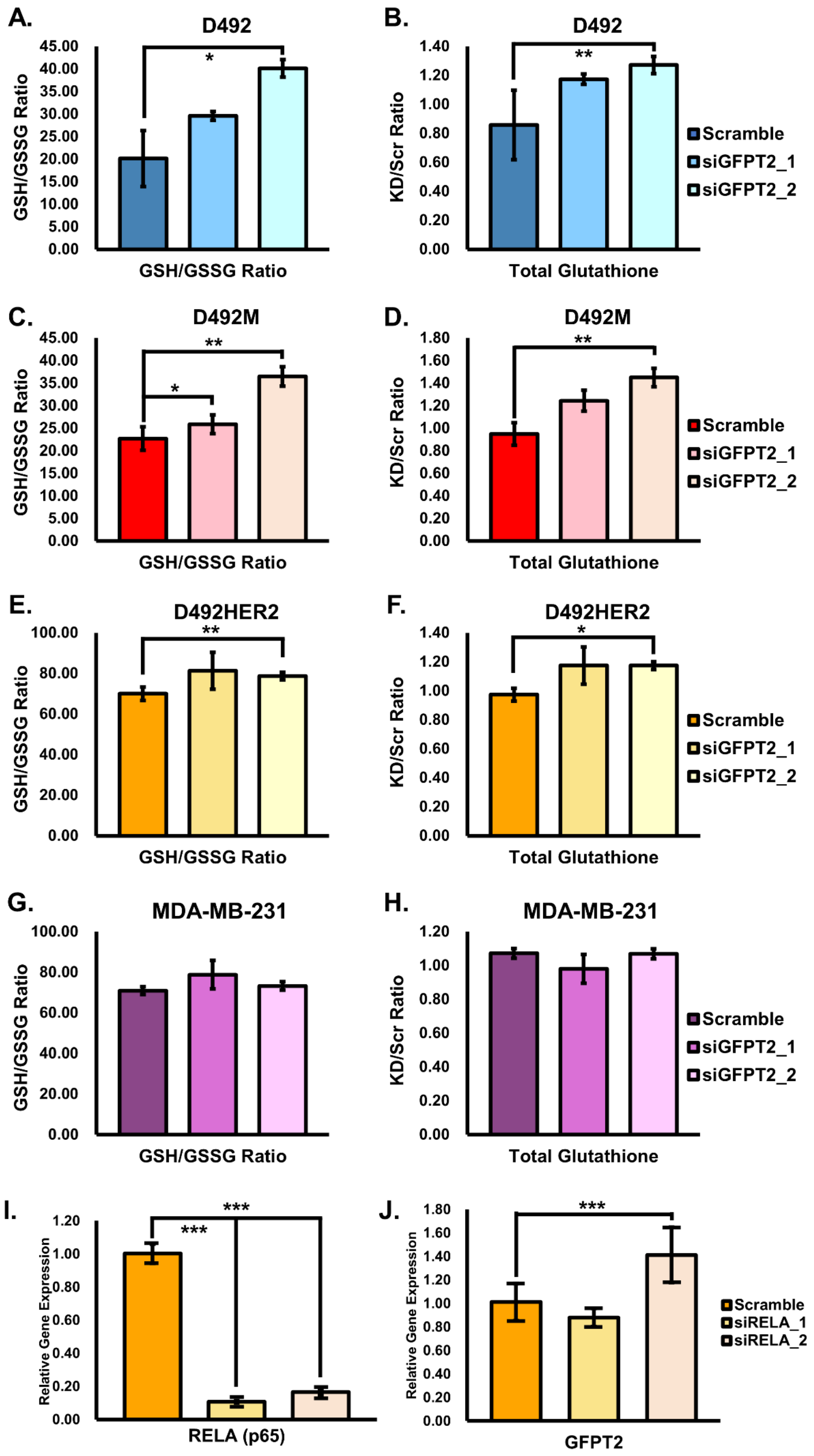
A.
D492
GSH/GSSG Ratio
GSH/GSSG Ratio
B.
D492
KD/Scr Ratio
Total Glutathione
Scramble
siGFPT2_1
siGFPT2_2
C.
D492M
GSH/GSSG Ratio
GSH/GSSG Ratio
D.
D492M
KD/Scr Ratio
Total Glutathione
E.
D492HER2
GSH/GSSG Ratio
GSH/GSSG Ratio
F.
D492HER2
KD/Scr Ratio
Total Glutathione
G.
MDA-MB-231
GSH/GSSG Ratio
GSH/GSSG Ratio
H.
MDA-MB-231
KD/Scr Ratio
Total Glutathione
I.
Relative Gene Expression
RELA (p65)
J.
Relative Gene Expression
GFPT2
Scramble
siRELA_1
siRELA_2

Figure S9. **Glutathione levels with *GFPT2* knockdown and GFPT2 RNA expression after siRNA-mediated knockdown of *NF-κB* (p65).** GSH/GSSG ratios and total glutathione levels after knockdown of *GFPT2* with two siRNAs in D492 **(A-B)**, D492M **(C-D)**, D492HER2 **(E-F)**, and MDA-MB-231 **(G-H)**. Increase of GSH/GSSG ratio and total glutathione level after knockdown of *GFPT2* with the second siRNA in all three D492 cell lines was observed. No significant differences in glutathione level were detected with *GFPT2* knockdown in MDA-MB-231. **(I)** The two siRNAs targeting *NF-κB* (p65) showed more than 80 % knockdown efficiency. **(J)** GFPT2 RNA expression was unchanged or increased after knockdown of *NF-kB* (p65, RELA) in D492HER2 with two siRNAs. RELA: the gene encodes NF-κB (p65). *****: $p < 0.05$; ******: $p < 0.01$; *******: $p < 0.001$. GSH: Reduced glutathione; GSSG: Oxidized glutathione; RELA: Proto-Oncogene, NF-κB subunit, transcription factor p65; GFPT2: Glutamine-fructose-6-phosphate transaminase 2.

Supplemental Tables

Table S1. **Primers in this study.**

Genes	Primers	Sequences(5'to3')
GFPT2	Forward	ATCCTTGCTTCGCCAAATGC
	Reverse	TTCAGTATCGTCCTTGGAGCAC
GALE	Forward	TTAGGGCTGGACAGGATGTGTG
	Reverse	CTGCTGCTTTTCCTGGTCCTTG
UGDH	Forward	TTTCTGTGCTGTCCAACCCTGA
	Reverse	CTCTCTGGCCCTCTGGAGTTTC
PGM2L1	Forward	GGGATCTGAACTCCAACCTGCT
	Reverse	AAAGACGATCTCGCAGCTCCTT
CDH1	Forward	ACCACGTACAAGGGTCAGGT
	Reverse	GGCATCAGCATCAGTCACTT
CDH2	Forward	CCTGCTTATCCTTGTGCTGA
	Reverse	CCTGGTCTTCTTCTCCTCCA
SQOR	Forward	CTTCAGGAAGACAGGGAAGCGA
	Reverse	TAACAGTGAGGTTCCGCTCCTG
GSK3B	Forward	GGCAGCAAGGTAACCACAGT
	Reverse	GATGGCAACCGATTCTCCAG
RELA (p65)	Forward	CCAGACCAACAACAACCCCT
	Reverse	TCACTCGGCAGATCTTGAGC
ACTB	Forward	CTTCCTGGGTGAGTGGAGACTG
	Reverse	GAGGGAAATGAGGGCAGGACTT

Table S2. **Summary of the LFQ and SILAC proteomic datasets.** Proteins were considered as valid quantification when at least two out of three replicates in one cell line were reported with valid intensities. Protein signature: a list of proteins up- or down-regulated in one cell line compared to the other two cell lines.

		LFQ	SILAC		
**Fractionation**		single shot	10 fractions		
**Replicates (per cell line)**		3	3		
**Peptides identified**		28,766	67,118 (Replicate 1)	70,314 (Replicate 2)	68,645 (Replicate 3)
**Total protein groups**		3,595	7,166 (Replicate 1)	7,390 (Replicate 2)	7,317 (Replicate 3)
**Quantifiable protein detected in at least two out of three replicates (Proteins were considered as valid and used for later analysis)**		2,705	5,120		
**D492 vs D492M (Unique proteins)**	Proteins only identified in D492	104	n.a		
	Proteins only identified in D492M	142	n.a		
**D492HER2 vs. D492 (Unique proteins)**	Proteins only identified in D492HER2	129	n.a		
	Proteins only detected in D492	123	n.a		
**D492HER2 vs. D492M (Unique proteins)**	Proteins only identified in D492HER2	76	n.a		
	Proteins only identified in D492M	108	n.a		
**D492 vs. D492M vs. D492HER2 (Unique proteins)**	Proteins only identified in D492	59	n.a		
	Proteins only identified in D492M	44	n.a		
	Proteins only identified in D492HER2	31	n.a		
**Protein signatures of all cell lines (Supplementary Data 3)**	D492 cell line signature	312 (210 downregulated and 102 upregulated)			
	D492M cell line signature	97 (49 downregulated and 48 upregulated)			
	D492HER2 cell line signature	84 (19 downregulated and 65 upregulated)			
